# mTORC2 mediates structural plasticity in distal nociceptive endings that contributes to pain hypersensitivity following inflammation

**DOI:** 10.1172/JCI152635

**Published:** 2022-08-01

**Authors:** Calvin Wong, Omer Barkai, Feng Wang, Carolina Thörn Perez, Shaya Lev, Weihua Cai, Shannon Tansley, Noosha Yousefpour, Mehdi Hooshmandi, Kevin C. Lister, Mariam Latif, A. Claudio Cuello, Masha Prager-Khoutorsky, Jeffrey S. Mogil, Philippe Séguéla, Yves De Koninck, Alfredo Ribeiro-da-Silva, Alexander M. Binshtok, Arkady Khoutorsky

**Affiliations:** 1 Department of Anaesthesia, McGill University, Montreal, Quebec, Canada.; 2 Department of Medical Neurobiology, Institute for Medical Research Israel-Canada, Faculty of Medicine, and; 3 The Edmond and Lily Safra Center for Brain Sciences, The Hebrew University, Jerusalem, Israel.; 4 F.M. Kirby Neurobiology Center, Boston Children’s Hospital and Harvard Medical School, Boston, Masschusetts, USA.; 5 Department of Psychiatry and Neuroscience, CERVO Brain Research Centre, Université Laval, Quebec City, Quebec, Canada.; 6 Department of Psychology,; 7 Department of Pharmacology and Therapeutics,; 8 Department of Physiology,; 9 Alan Edwards Centre for Research on Pain,; 10 Montreal Neurological Institute, Department of Neurology and Neurosurgery,; 11 Department of Anatomy and Cell Biology, and; 12 Faculty of Dental Medicine and Oral Health Sciences, McGill University, Montreal, Quebec, Canada.

**Keywords:** Neuroscience, Mouse models, Pain, Signal transduction

## Abstract

The encoding of noxious stimuli into action potential firing is largely mediated by nociceptive free nerve endings. Tissue inflammation, by changing the intrinsic properties of the nociceptive endings, leads to nociceptive hyperexcitability and thus to the development of inflammatory pain. Here, we showed that tissue inflammation–induced activation of the mammalian target of rapamycin complex 2 (mTORC2) triggers changes in the architecture of nociceptive terminals and leads to inflammatory pain. Pharmacological activation of mTORC2 induced elongation and branching of nociceptor peripheral endings and caused long-lasting pain hypersensitivity. Conversely, nociceptor-specific deletion of the mTORC2 regulatory protein rapamycin-insensitive companion of mTOR (Rictor) prevented inflammation-induced elongation and branching of cutaneous nociceptive fibers and attenuated inflammatory pain hypersensitivity. Computational modeling demonstrated that mTORC2-mediated structural changes in the nociceptive terminal tree are sufficient to increase the excitability of nociceptors. Targeting mTORC2 using a single injection of antisense oligonucleotide against Rictor provided long-lasting alleviation of inflammatory pain hypersensitivity. Collectively, we showed that tissue inflammation–induced activation of mTORC2 causes structural plasticity of nociceptive free nerve endings in the epidermis and inflammatory hyperalgesia, representing a therapeutic target for inflammatory pain.

## Graphical abstract

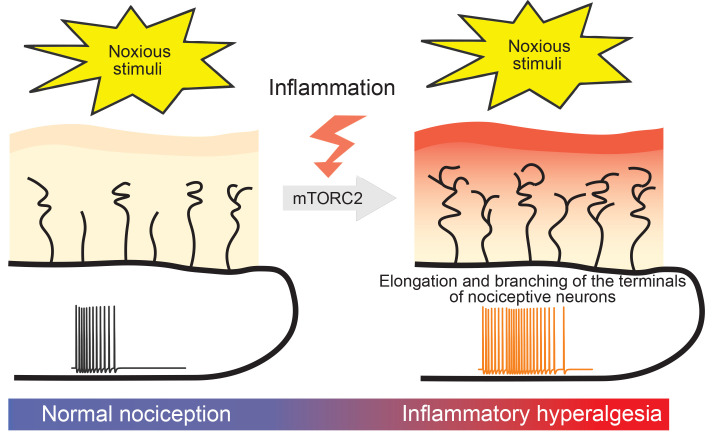

## Introduction

Chronic pain is a common (~20% of the world population) debilitating condition that can persist for years, severely affecting the quality of life of affected individuals and their families (1). The current available treatments for chronic pain are of limited efficacy and can often lead to severe side effects, such as addiction. A better understanding of the cellular and molecular mechanisms of chronic pain is essential to developing non–opioid-based therapeutics that target the processes underlying the development of maladaptive persistent pain.

Sensitization of nociceptive primary efferent fibers following tissue inflammation is a critical mechanism of inflammatory pain. Proinflammatory mediators released following inflammation act on the nociceptive terminals in the epidermis. They affect the encoding of noxious stimuli by modulating the activity of transducer channels (such as TRPV1) and voltage-gated channels, leading to nociceptor hyperexcitability. Despite progress in understanding molecular mechanisms underlying nociceptive sensitization, it remains unknown whether, in addition to the changes in nociceptive terminals’ intrinsic properties, inflammation also promotes nociceptive structural plasticity that contributes to nociceptor hyperexcitability and hyperalgesia.

**Figure 1:**
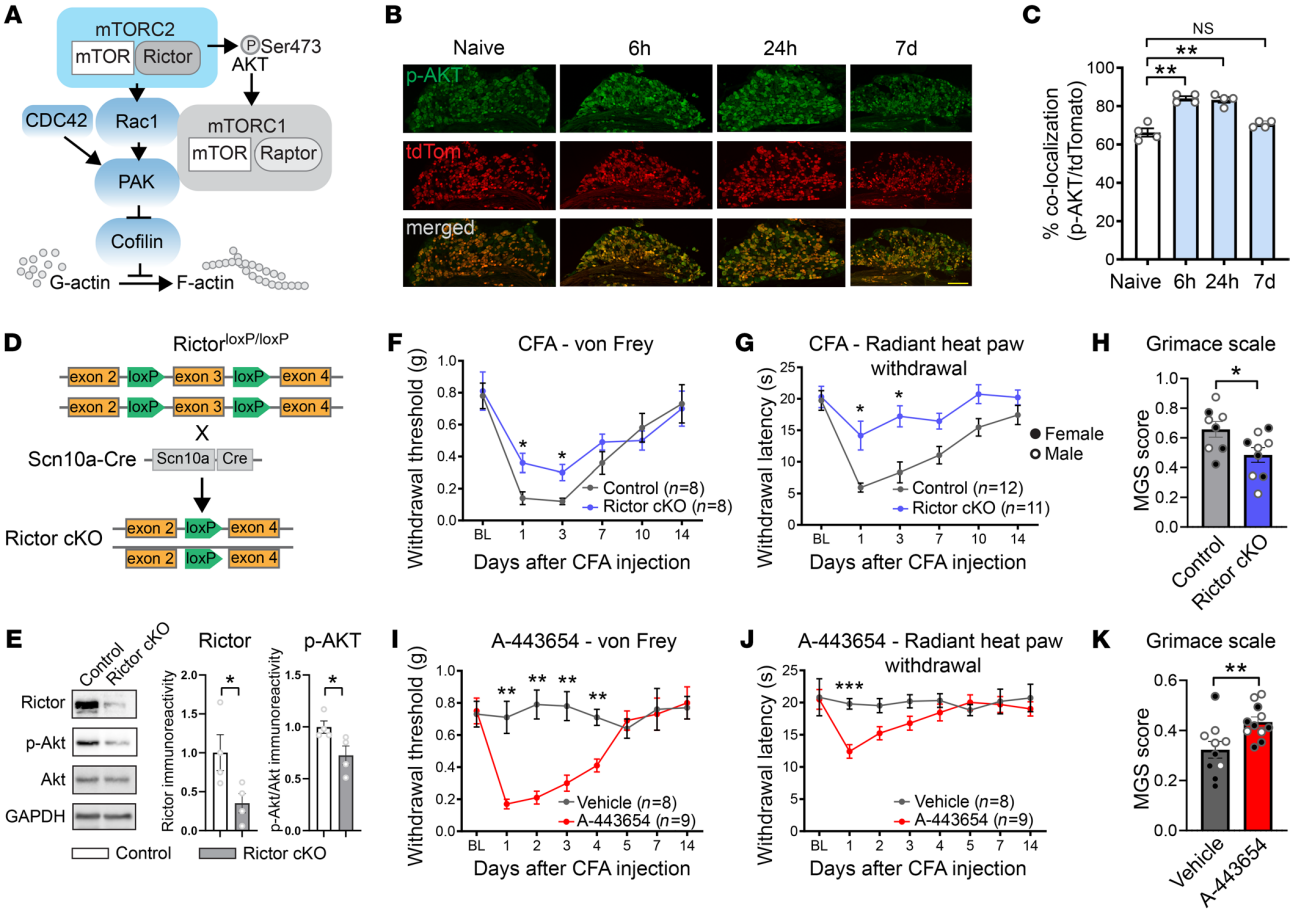
Nociceptor-specific deletion of Rictor alleviates inflammation-induced pain. (**A**) Schematic of mTORC2 signaling. (**B**) p-Akt (Ser473) immunostaining of DRG from tdTomato;*Scn10a^Cre^* naive mice and at 6 hours, 24 hours, and 7 days after intraplantar injection of CFA. Scale bar: 200 μm. (**C**) Quantification (*n =* 4 mice per group, 1-way ANOVA followed by Bonferroni’s post hoc comparison). (**D**) Generation of Rictor-cKO mouse. (**E**) Western blots showing decreased Rictor and p-Akt (S473) in DRG lysates (*n =* 4 mice per group, Student’s *t* test, 2 tailed). Intraplantar injection of CFA induces reduced mechanical (**F**) and thermal (**G**) hypersensitivity in Rictor-cKO mice compared with control (Rictor^fl/fl^ and Scn10a^Cre^) animals (2-way ANOVA followed by Bonferroni’s post hoc comparison). (**H**) Reduced spontaneous pain in Rictor-cKO mice 1 day after intraplantar injection of CFA (Student’s *t* test 2 tailed). Intraplantar injection of A-443654 (20 μL, 8 mM) induces mechanical (**I**) and thermal (**J**) hypersensitivity (2-way ANOVA followed by Bonferroni’s post hoc comparison). (**K**) Intraplantar injection of A-443654 causes spontaneous pain 1 day after injection (Student’s *t* test, 2 tailed). All data are represented as mean ± SEM. *P < 0.05; **P < 0.01; ***P < 0.001.

The evolutionarily conserved kinase mammalian target of rapamycin (mTOR) exists in 2 functionally and structurally distinct multiprotein complexes. The better-studied complex, mTOR complex 1 (mTORC1), is defined by the regulatory protein Raptor and is characterized by its sensitivity to rapamycin (2). mTORC1 has been extensively studied in the field of pain in the context of its primary function of regulating the rate of mRNA translation (3). mTORC2, defined by rapamycin-insensitive companion of mTOR (Rictor) (4), plays a central role in synaptic plasticity and memory (5). Specifically, mTORC2 is required for the late phase of long-term potentiation (L-LTP) (6), long-term depression (LTD) (7), and memory formation, acting by regulating actin-dependent dendritic spine plasticity in hippocampal neurons (6–8). Whether activation of mTORC2 is involved in pain-related plasticity is unknown. mTORC2 plays a key role in regulating the conversion of globular actin (G-actin) to polymerized filamentous actin (F-actin) via downstream signaling to the Rho GTPase Rac1 and its subsequent effectors, PAK1 and cofilin (9) (Figure 1A). A secondary effector of mTORC2 is phosphorylated Akt at Ser473, which is the most reliable readout of mTORC2 activity (10).

Previous studies have reported a tissue inflammation–induced increase in Akt phosphorylation at Ser473 in nociceptors (11, 12), indicating enhanced mTORC2 activity. However, the functional significance of mTORC2 activation in nociception and pain has not been examined. In this study, we investigated whether mTORC2 plays a role in the development of pain hypersensitivity in response to tissue inflammation. We found that tissue inflammation that causes hyperalgesia induces growth and increases the complexity of nociceptive distal endings in the glabrous skin epidermis and cornea. Importantly, these structural changes and inflammation-mediated hyperalgesia were prevented by knocking out the key component of mTORC2, *Rictor*, selectively in Na_v_1.8-expressing nociceptors. Activation of mTORC2 with a single injection of the small molecule A-443654 (13) led to long-lasting pain hypersensitivity in naive animals and induced the growth and branching of nociceptive terminals. In summary, we demonstrate that mTORC2 activation is an important driver of inflammatory pain and propose that mTORC2-dependent hypersensitivity is mediated via structural changes in nociceptive free nerve endings.

## Results

### mTORC2 is activated in nociceptors during tissue inflammation.

To establish whether mTORC2 is activated in nociceptors in response to tissue inflammation, we subjected mice to a model of inflammation evoked by intraplantar injection of CFA and measured the phosphorylation of Akt (p-Akt) at Ser473, which is the most reliable readout of mTORC2 activity (10) (Figure 1A). To examine mTORC2 activation in nociceptive neurons, we used mice expressing tdTomato under the control of the nociceptor-specific *Scn10a* (Na_v_1.8) promoter (tdTomato *Scn10a*^Cre^) (14). Consistent with previous reports (11, 12), we found a significant increase in the number of Na_v_1.8 tdTomato-positive DRG neurons with elevated Akt phosphorylation 6 and 24 hours after CFA injection (Figure 1, B and C), indicating activation of mTORC2 in nociceptors following CFA-induced inflammation.

### Genetic inhibition of mTORC2 alleviates inflammation-induced hyperalgesia.

To study the functional role of mTORC2 activation in the development of pain, we generated transgenic mice with the deletion of the gene encoding Rictor, a defining component of the mTORC2 complex (Figure 1A), selectively in nociceptors. *Rictor* floxed mice were crossed with mice expressing Cre recombinase under the control of the *Scn10a* (Na_v_1.8) promoter (*Rictor^loxP/loxP^* *Scn10a*^Cre^), hereafter referred to as *Rictor* conditional knockout (Rictor-cKO) mice (Figure 1D). As expected, Rictor-cKO mice showed reduced expression of Rictor and p-Akt (Ser473) in DRG lysates (Figure 1E). Behavioral studies demonstrated that Rictor-cKO mice exhibited no change in baseline mechanical sensitivity, as measured in von Frey and tail-clip assays (Supplemental Figure 1, A and B; supplemental material available online with this article; https://doi.org/10.1172/JCI152635DS1), and showed no alterations in basal thermal sensitivity, as assessed in the radiant heat paw-withdrawal, tail-flick, and hot-plate assays (Supplemental Figure 1, C–E). No changes in the proportion of major cell populations (IB4^+^, CGRP^+^, and NF200^+^) were observed in the DRG of Rictor-cKO mice as compared with control animals (Supplemental Figure 1, F–H).

Importantly, intraplantar injection of CFA in Rictor-cKO mice led to significantly reduced mechanical and thermal hypersensitivity (Figure 1, F and G; analysis for each sex separately in Supplemental Figure 2, A–D) and spontaneous pain (assessed using the mouse grimace scale [MGS]; Figure 1H) compared with control animals. Pain hypersensitivity produced by intraplantar injection of another inflammogen, γ-carrageenan, was also reduced in Rictor-cKO mice (Supplemental Figure 2, E and F). Paw edema was not different in the 2 genotypes in both CFA and γ-carrageenan assays (Supplemental Figure 2, G and H). These results indicate that ablation of Rictor in nociceptive neurons attenuates inflammatory pain. No differences in the development of nerve injury–induced hypersensitivity were found between Rictor-cKO and control mice in 2 models of neuropathic pain, spared nerve injury (SNI) (Supplemental Figure 2I) and chronic constriction injury (CCI) (Supplemental Figure 2, J and K). Together, these results indicate that mTORC2 is activated in nociceptors in response to tissue inflammation and that its genetic ablation reduces inflammatory but not neuropathic pain.

### Pharmacological activation of mTORC2 causes pain hypersensitivity.

To study whether activation of mTORC2 induces pain hypersensitivity, we injected mice with a small molecule activator of mTORC2, A-443654 (13). A single i.p. injection of A-443654 led to long-lasting mechanical and thermal hypersensitivity (Supplemental Figure 3, A and B). To establish whether peripheral activation of mTORC2 is sufficient to produce hypersensitivity, we injected A-443654 locally into the mouse hind paw. A single intraplantar injection of A-443654 induced long-lasting mechanical and thermal hypersensitivity (Figure 1, I and J; analysis for each sex separately in Supplemental Figure 3, C-F) and triggered spontaneous pain (MGS at day 1 after A-443654 injection; Figure 1K). The hyperalgesic effect of A-443654 was not affected by mTORC1 inhibitor CCI-779 (Supplemental Figure 3, G and H), indicating that the effect of A-443654 on hypersensitivity is not dependent on mTORC1.

### Tissue inflammation promotes elongation and branching of nociceptive terminal fibers in the glabrous paw skin.

**Figure 2:**
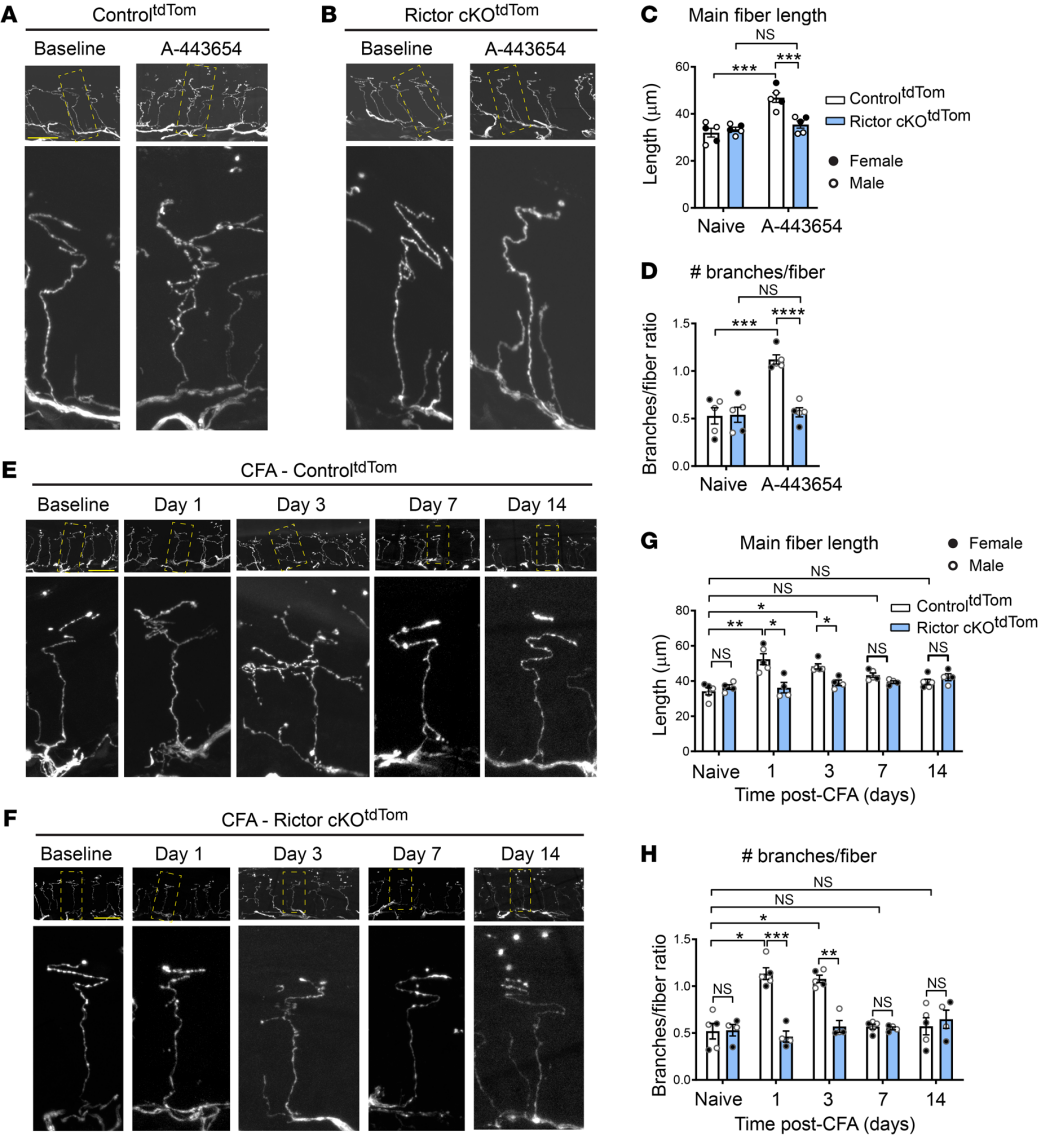
Activation of mTORC2 engenders structural remodelling of nociceptive distal endings in epidermis. A-443654 (20 μL of 8 mM, intraplantar) was injected in Control^tdTom^ (**A**) and Rictor-cKO^tdTom^ (**B**) mice, and glabrous skin from the injected paw was collected 24 hours later. Representative images of the low (top) and high (bottom) magnification of nociceptive fibers in the epidermis, acquired using confocal microscopy with AiryScan. A-443654 increases main fiber length in Control^tdTom^ but not Rictor-cKO^tdTom^ mice (**C**, *n =* 5 mice per group). Similarly, A-443654 increases the number of branches per fiber in Control^tdTom^ but not Rictor-cKO^tdTom^ mice (**D**, *n =* 5 mice per group). Control^tdTom^ (**E**) and Rictor-cKO^tdTom^ (**F**) mice were injected with CFA (intraplantar), and glabrous skin from the injected paw was collected at day 1, day 3, day 7, and day 14 after injection. CFA increases main fiber length in Control^tdTom^ but not Rictor-cKO^tdTom^ mice at day 1 after CFA (**G**, *n =* 4–5 per group). Similarly, CFA increases the number of branches per fiber in Control^tdTom^ but not Rictor-cKO^tdTom^ mice (**H**, *n =* 4–5 per group). Statistics are based on 2-way ANOVA followed by Bonferroni’s post hoc comparison. All data are represented as mean ± SEM. *P < 0.05; **P < 0.01; ***P < 0.001; ****P < 0.0001. Scale bars: 20 μm.

To study the mechanism by which activation of mTORC2 promotes pain, we focused on its primary functions, namely the regulation of actin dynamics and structural plasticity. To assess morphological changes in nociceptor terminals in response to mTORC2 activation, we injected control and Rictor-cKO mice expressing tdTomato in nociceptors (tdTomato;*Scn10a^Cre^*, abbreviated as Control^tdTom^; Rictor^fl/fl^;tdTomato;*Scn10a^Cre^*, abbreviated as Rictor-cKO^tdTom^) with the mTORC2 activator A-443654 intraplantarly. Glabrous skin tissue was collected 24 hours after injection, and skin sections were imaged using high-resolution confocal microscopy with AiryScan (Figure 2, A and B). Morphological analysis of nociceptors in the epidermis of Control^tdTom^ mice, reconstructed from serial optical sections, revealed a significant increase in the length of terminal nociceptive fibers (Figure 2C) and an increased number of branch-like distal structures protruding from these fibers (Figure 2D). No changes in the number of fibers (Supplemental Figure 4A), branch length (Supplemental Figure 4, B and C), or distance to branching point (Supplemental Figure 4, B and D) were found. Intraplantar injection of A-443654 in Rictor-cKO^tdTom^ mice failed to cause distal fiber growth and branching of the nociceptive fibers (Figure 2, B–D). These results show that the activation of mTORC2 is sufficient to induce structural changes in nociceptor terminal endings and highlight the specificity of A-443654 for mTORC2.

We next investigated whether CFA-induced tissue inflammation causes structural changes in the nociceptive neurons innervating the epidermis and studied the role of mTORC2 in this process. In Control^tdTom^ mice, nociceptive terminal architecture was changed at the time points that correspond with the highest CFA-mediated hypersensitivity (1 and 3 days after CFA injection), but not on days 7 and 14, when pain hypersensitivity subsides. On days 1 and 3, nociceptive terminals in the epidermis possessed elongated distal endings and an increased number of branches on the main fiber (Figure 2, E–H). No changes were found in the number of fibers (Supplemental Figure 4E), branch length (Supplemental Figure 4F), or distance to the branching point (Supplemental Figure 4G). Notably, CFA injection did not produce any changes in the nociceptor’s structure in Rictor-cKO^tdTom^ mice (Figure 2, F–H). Thus, peripheral inflammation induces structural hyperinnervating changes in the nociceptive peripheral terminals via mTORC2.

Nociceptive fibers in the mouse glabrous skin can be broadly classified as peptidergic and nonpeptidergic, exhibiting different expression of ion channels (e.g., less Na_v_1.9 TTX-resistant sodium channels in peptidergic fibers) (15, 16), morphology (peptidergic fibers are shorter and terminate in stratum spinosum, whereas nonpeptidergic fibers terminate in stratum granulosum) (17), and function (peptidergic fibers predominantly mediate noxious heat, whereas nonpeptidergic fibers mediate mechanical sensation) (18, 19). To assess structural plasticity in each subpopulation, we immunostained Na_v_1.8-tdTomato nociceptive fibers for CGRP to identify peptidergic (CGRP^+^) and nonpeptidergic (CGRP^–^) fibers. CFA and A-443654 induced morphological changes (increase in length of the main fiber and the number of branches per fiber) in both peptidergic and nonpeptidergic fibers, and these changes were abolished in Rictor-cKO mice (Supplemental Figure 5, A–F).

**Figure 3:**
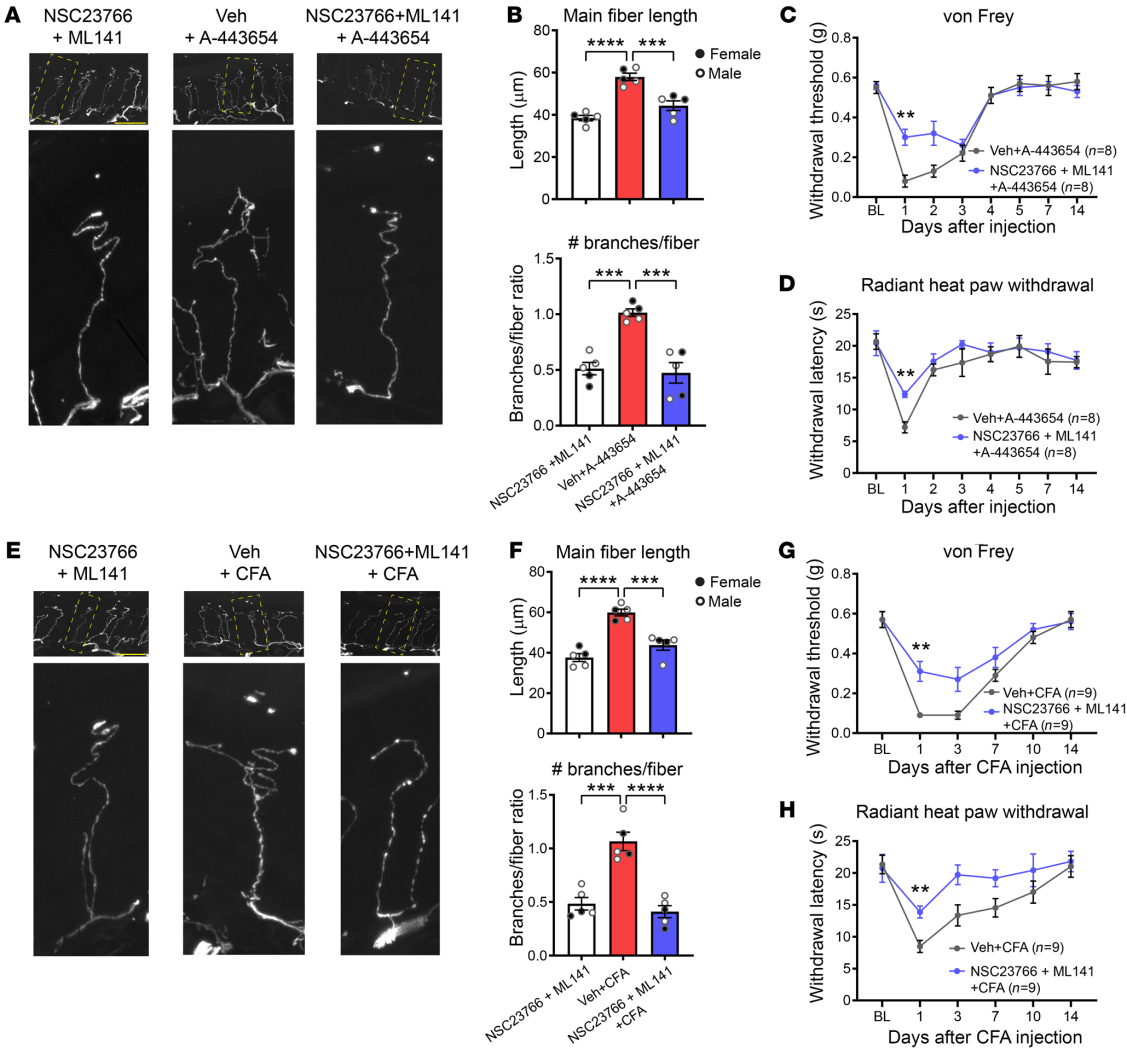
mTORC2 promotes structural changes in distal nociceptive endings and pain via modulation of actin dynamics. Control^tdTom^ mice were injected with A-443654 alone (intraplantar, 20 μL, 8 mM) and together with an inhibitor of Rac1 (NSC23766, intraplantar, 10 μL, 10 mM, 15 minutes prior to A-443654) and CDC42 (ML141, intraplantar, 10 μL, 50 μM, 15 minutes prior to A-443654). Imaging of tissue collected 24 hours after A-443654 injection revealed that Rac1/CDC42 inhibitors prevent A-443654–induced structural changes in nociceptive endings in epidermis (**A**). Quantification shows that NSC23766/ML141 block A-443654–induced increase in main fiber length (**B**, top, *n =* 5 mice per group) and the number of branches per fiber (**B**, bottom). NSC23766/ML141 also alleviated A-443654–induced hypersensitivity (**C** and **D**). (**E**) Mice were injected with CFA (intraplantar) in the absence and presence of NSC23766/ML141 (intraplantar, 15 minutes prior to CFA), and the glabrous skin from the injected paw was fixed 24 hours later and imaged. NSC23766/ML141 blocked CFA-induced increase in main fiber length (**F**, top, *n =* 5 mice per group) and the number of branches per fiber (**F**, bottom). NSC23766/ML141 also alleviated CFA-induced hypersensitivity (**G** and **H**). One-way ANOVA followed by Bonferroni’s post hoc comparison (**B** and **F**); 2-way ANOVA followed by Bonferroni’s post hoc comparison (**C**, **D**, **G**, and **H**). All data are represented as mean ± SEM. **P < 0.01; ***P < 0.001; ****P < 0.0001. Scale bars: 20 μm.

mTORC2 is thought to mediate structural plasticity via its effect on Rho-GTPases Rac1 and CDC42 (Ras-related C3 botulinum toxin substrate 1), which promote PAK and cofilin phosphorylation to stimulate actin dynamics. To determine whether mTORC2 activation produces structural changes and pain via Rac1/CDC42, we performed intraplantar A-443654 injection while locally inhibiting Rac1 and CDC42 by administration of their specific inhibitors NSC23766 and ML141, respectively. Injection of Rac1 and CDC42 inhibitors 15 minutes prior to A-443654 administration prevented mTORC2-induced nociceptor structural changes (Figure 3, A and B) and alleviated hypersensitivity (Figure 3, C and D). Importantly, Rac1 and CDC42 inhibition also prevented CFA-induced distal fiber growth and branching (Figure 3, E and F) and reduced CFA-induced hypersensitivity (Figure 3, G and H). Together, these results show that tissue inflammation leads to hyperinnervating nociceptive structural plasticity and hyperalgesia via mTORC2-mediated Rac1/CDC42 activity.

### Ablation of mTORC2 inhibits inflammation-induced corneal nociceptor structural changes and pain.

**Figure 4:**
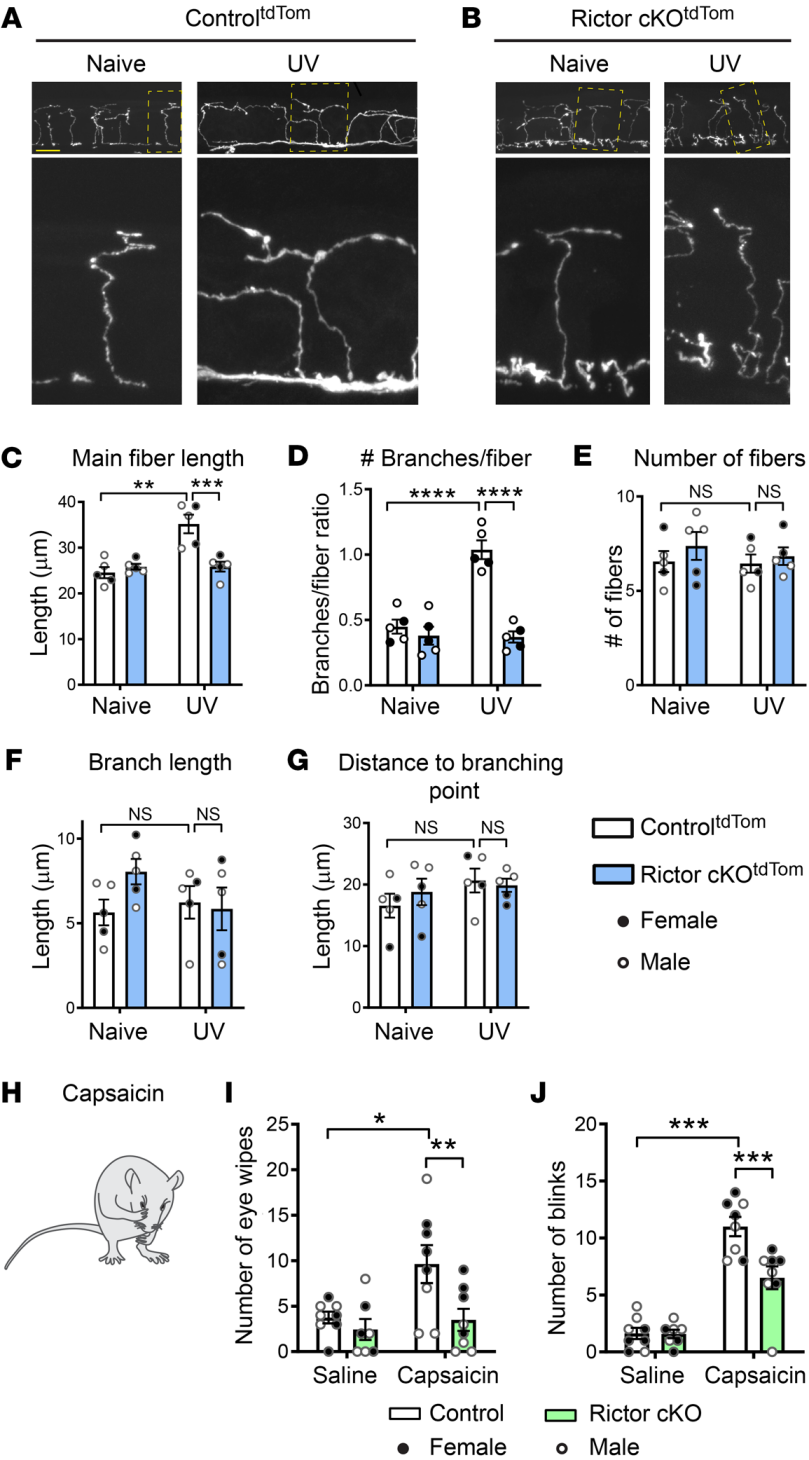
mTORC2 mediates inflammation-induced structural changes in nociceptive fibers in cornea and promotes pain. Control^tdTom^ and Rictor-cKO^tdTom^ mice were subjected to UV irradiation (22 minutes, 450 mJ/cm^2^), and cornea tissue was extracted 24 hours later. Confocal imaging shows that UV-induced inflammation promotes structural changes in control mice (**A**), but not in Rictor-deficient animals (**B**). UV induces changes in Control^tdTom^ but not in Rictor-cKO^tdTom^ mice in main fiber length (**C**) and the number of branches per fiber (**D**). No differences were seen between naive and UV-irradiated mice in the number of fibers (**E**), branch length (**F**), or distance to branching point (**G**) (*n =* 5 animals per group for **A**–**G**, 2-way ANOVA followed by Bonferroni’s post hoc comparison). Twenty-four hours after UV irradiation, capsaicin (10 μl of 1 μM) was applied unilaterally onto the eye (**H**) and the numbers of wipes (**I**) and blinks (**J**) within 5 minutes after administration were quantified (2-way ANOVA mixed effects model followed by Bonferroni’s post hoc comparison). All data are represented as mean ± SEM. *P < 0.05; **P < 0.01; ***P < 0.001; ****P < 0.0001. Scale bar: 10 μm.

To study the role of mTORC2 in inflammation-induced structural plasticity of nociceptive peripheral terminals and inflammatory pain in a different functional environment, namely the cornea, where fiber arrangement and cellular composition surrounding the terminals are different from glabrous skin (20), we employed a model of corneal inflammation produced by UV irradiation (21). Corneal sections from Control^tdTom^ and Rictor-cKO^tdTom^ mice were collected 24 hours following UV irradiation (22 minutes, 450 mJ/cm^2^). Morphological analyses showed that corneal inflammation induces changes in the nociceptor terminal structure similar to what we observed in the inflamed epidermis. UV photokeratitis led to increased nociceptor nerve ending length and branching in Control^tdTom^ mice, but not in Rictor-cKO^tdTom^ animals (Figure 4, A–D). No changes in the number of fibers (Figure 4E), branch length (Figure 4F), and distance to branching point (Figure 4G) were found. Importantly, capsaicin-induced eye wiping and blinking behaviors (21) observed in UV photokeratitis in control mice were significantly reduced in Rictor-cKO mice (Figure 4, H–J). Thus, similarly to what occurs with the glabrous skin, mTORC2 activation in corneal nociceptors contributes to inflammation-induced nociceptive structural plasticity and pain.

### mTORC2-mediated structural changes increase neuronal excitability.

We next asked whether the mTORC2-mediated structural changes in nociceptive terminals could underlie mTORC2-induced hyperalgesia. The effects of the nociceptive terminals’ architecture on their input-output characteristics were recently suggested (22), showing that an increase in the length of the terminal ending leads to a substantial increase in firing of modeled nociceptive neurons (22). Moreover, the model predicts that by allowing voltage summation, an increased number of terminal branches amplifies the excitability of the modeled neuron (22). Accordingly, we hypothesized that inflammation-induced and mTORC2-mediated elongation of terminal endings and the increase in branching could be sufficient to enhance nociceptive excitability.

To rule out the possibility that mTORC2 activation promotes hypersensitivity via its direct effect on neuronal excitability, we performed several experiments. First, we cultured DRG neurons from tdTomato;*Scn10a^Cre^* mice and measured membrane passive and active properties of tdTomato^+^ nociceptors before and after the administration of A-443654 (Supplemental Figure 6A). We confirmed that A-443654 (1 μM for 10 minutes) activates mTORC2, as evidenced by increased p-Akt (Supplemental Figure 6, B and C). Recording from tdTomato^+^ nociceptive neurons showed that A-443654 does not change the resting membrane potential (RMP), input resistance, action potential (AP) rheobase and threshold, number of APs evoked by ramp stimulation, AP amplitude, and AP half-width (Supplemental Figure 6, D–J). We also injected tdTomato;*Scn10a^Cre^* mice with A-443654 (i.p.) or vehicle and prepared DRG cultures 24 hours later (Supplemental Figure 6K). No differences in the excitability of tdTomato^+^ nociceptive neurons were detected between the 2 groups (Supplemental Figure 6, L–R). Finally, we studied whether A-443654 affects the intrinsic excitability of Na_v_1.8-positive DRG neurons by performing in vivo calcium imaging. We accessed calcium responses of DRG nociceptors following electrical stimulation applied proximally to the terminal fibers to the sciatic nerve in anesthetized *Scn10a^Cre^*;GCaMP6s mice. A-443654–injected mice, which exhibited mechanical hypersensitivity (Supplemental Figure 6S), did not show increased calcium transients or number of activated neurons in response to sciatic nerve stimulation as compared with vehicle-injected animals (Supplemental Figure 6, T–W). Together, these experiments suggest that the activation of mTORC2 does not affect the intrinsic excitability of Na_v_1.8-positive DRG neurons. Additionally, no macroscopic changes were observed in the density and distribution of the central branch of nociceptive fibers labeled by tdTomato in the spinal cord dorsal horn (Supplemental Figure 7).

**Figure 5:**
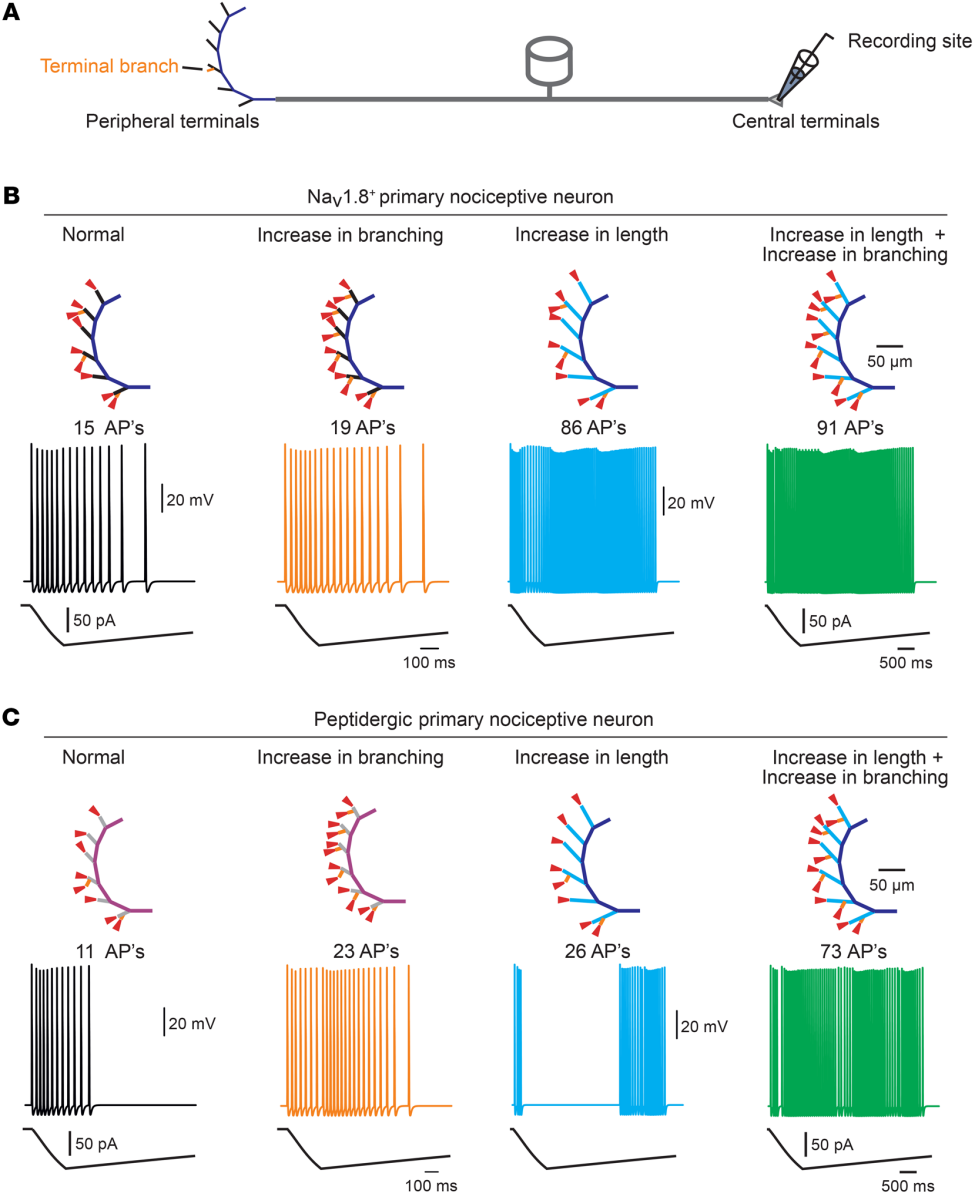
Structural changes in distal nociceptive endings are sufficient to produce hypersensitivity. (**A**) Schematic depicting the model of the primary nociceptive neuron. The peripheral terminal tree morphology is rendered from the structure of a terminal innervating the glabrous skin of the mouse paw in vivo. The stimulations are applied to the terminal branch ends (red triangles shown in **B**), and the recording electrode is placed at the central terminal. (**B**) Simulated current-clamp recordings from the central terminal following stimulation by a capsaicin-like current of all Na_v_1.8-positive nociceptor terminal branches in simulated normal conditions (black); simulated increase in branching (100%, orange); simulated increase in length of the terminal endings (from 32 μm to 46 μm, blue); and a combined increase in branching and terminal length (green). (**C**) Same as **B**, but applied to the modeled peptidergic Na_v_1.8-positive nociceptor terminal branches in simulated normal conditions (black); simulated increase in branching (200%, orange); simulated increase in length of the terminal endings (from 26 μm to 44.6 μm, blue); and a combined increase in branching and terminal length (green). Schematic depicting the simulated models in **B** and **C** are shown above.

We next determined whether mTORC2-mediated structural changes at the terminal fibers are sufficient to affect neuronal activity by employing a computational modeling approach. We utilized the numerical model of a nociceptive terminal tree we previously described (22) to predict how an increase in the distal ending length and terminal branching affects the response of the modeled nociceptive neuron to capsaicin-like stimuli. We implemented the changes in length and branching we found in vivo to a model representing all Na_v_1.8-expressing nociceptors (Figure 5, A and B) and to a model possessing the features of CGRP^+^ peptidergic nociceptors (Figure 5C, see Methods). We then stimulated the terminal endings with the simulated capsaicin-like current and examined the resulting AP firing at the central terminal. Our model predicts that the elongation of the terminal length and the increase in branching are sufficient to increase the firing of APs at the central terminals (Figure 5, B and C) in response to capsaicin-like stimulation. Moreover, we showed that structural changes in the nociceptive terminal tree led to changes in time pattern of firing (Supplemental Figure 8), suggesting that in addition to changes in the AP number that encodes for enhanced nociceptive output toward the CNS, there is also a change in timing, which may influence nociceptive neuronal processing (22). These data suggest that the mTORC2-induced increase in terminal growth and branching is sufficient to enhance the response of nociceptive neurons to noxious stimuli.

### Rictor-ASO therapy alleviates inflammatory pain.

**Figure 6:**
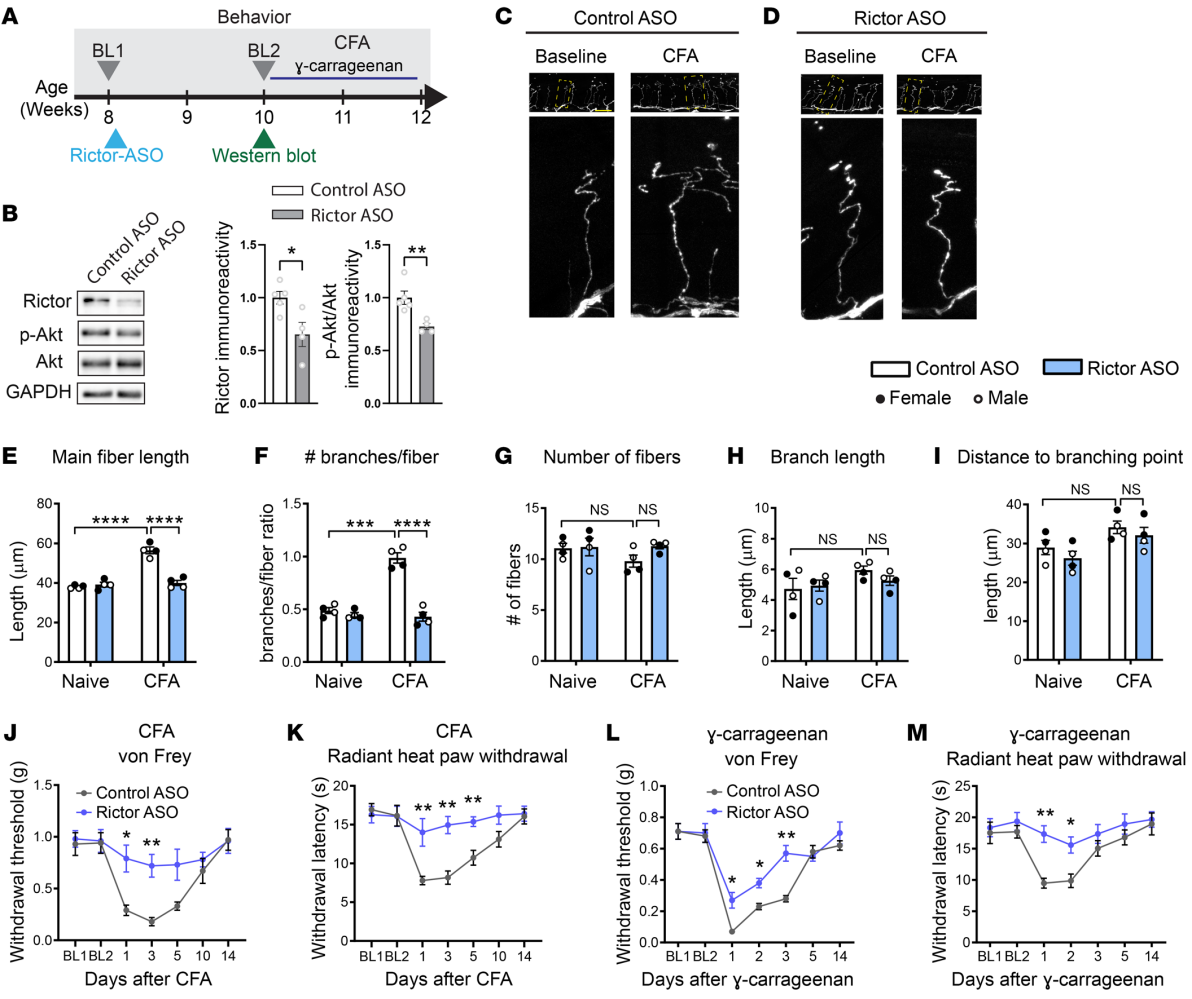
Antisense oligonucleotide targeting Rictor alleviates inflammatory pain. (**A**) Schematic shows experimental design of Rictor antisense oligonucleotide (Rictor-ASO) treatment (s.c.). (**B**) Western blots and quantification show that Rictor-ASO reduces the levels of Rictor and p-Akt (Ser473) (*n =* 4–5 mice per group, Student’s *t* test, 2 tailed). Control^tdTom^ mice were injected with Control- (**C**) and Rictor-ASO (**D**) (100 mg/kg s.c.) 2 weeks prior to intraplantar injection of CFA, and the glabrous skin from the injected paw was collected 24 hours later. Representative images of the low (top) and high (bottom) magnification of nociceptive fibers in the epidermis were acquired using confocal microscopy with AiryScan. Rictor-ASO rescued CFA-induced increases in main fiber length (**E**, *n =* 4 mice per group) and increases in the number of branches per fiber (**F**, *n =* 4 mice per group). No differences between Control-ASO and Rictor-ASO mice after CFA intraplantar injection in the number of fibers (**G**), branch length (**H**), or distance to branching point (**I**). Treatment with Rictor-ASO alleviates hypersensitivity induced by CFA (**J**, von Frey; **K**, radiant heat paw withdrawal) and γ-carrageenan (**L**, von Frey; **M**, radiant heat paw withdrawal). Two-way ANOVA followed by Bonferroni’s post hoc comparison. All data are represented as mean ± SEM. *P < 0.05; **P < 0.01; ***P < 0.001, ****P < 0.0001. Scale bar: 20 μm.

Our data underscore the role of mTORC2 activation in the development of inflammatory pain. Since there are no pharmacological inhibitors to disrupt mTORC2*,* we used a short synthetic ASO to target *Rictor* as a potential therapeutic avenue for alleviation of inflammatory pain. Rictor-ASO, which binds with exact complementarity to the mouse *Rictor* transcript (8), has been previously shown to specifically downregulate *Rictor* levels and inhibit mTORC2, but not mTORC1, activity (8). A single s.c. injection of Rictor-ASO reduced Rictor protein levels and inhibited mTORC2 in DRG lysates, as assessed by reduced p-Akt (Figure 6, A and B). The inhibition was not present in spinal cord lysates (Supplemental Figure 9). Remarkably, Rictor-ASO rescued CFA-induced morphological changes (increase in fiber length and number of branches per fiber) (Figure 6, C–I) and substantially decreased mechanical and thermal hypersensitivity induced by CFA (Figure 6, J and K) and γ-carrageenan (Figure 6, L and M), whereas Control-ASO had no effect. These findings show that a single injection of Rictor-ASO provides a therapeutic option for targeting mTORC2 in inflammatory pain.

## Discussion

In this study, we show that peripheral inflammation induces structural changes, including elongation and branching of distal endings of nociceptive fibers, and demonstrate that this process is controlled by mTORC2. We propose that mTORC2-mediated changes in nociceptive fiber architecture contribute to their increased excitability and the development of inflammatory pain.

Phosphorylation of Akt at Ser473, which reflects mTORC2 activation, has been repeatedly documented in numerous models of pain, including acute and chronic inflammatory pain (11, 12, 23), neuropathic pain (23, 24), and cancer-induced pain (25). Moreover, consistent with our results, increased Akt phosphorylation at Ser473 in response to stimulation was specifically detected in small-diameter nociceptors (11, 12). Uncovering the functional role of mTORC2 activation in the development of pain, however, has been hindered by embryonic lethality of Rictor whole-body KO mice (26) and the lack of pharmacological tools to inhibit mTORC2.

Based on previous studies (11, 12, 23) and our results showing mTORC2 activation in nociceptors, we focused on the role of mTORC2 in Na_v_1.8^+^ nociceptors. Conditional ablation of Rictor, a central component of mTORC2, in nociceptors alleviated the development of inflammatory pain. It also prevented inflammation-induced structural changes in nociceptive peripheral terminals in the epidermis. Several lines of evidence support the notion that mTORC2-mediated changes in nociceptor distal ending architecture contribute to hypersensitivity. First, mTORC2 activation-induced hypersensitivity was alleviated by inhibition of actin dynamic upstream signaling, demonstrating the contribution of structural actin-mediated mechanisms to the effect of mTORC2 activation on pain. Second, the time line of the effect of a single intraplantar injection of mTORC2 activator, A-443654, on mechanical sensitivity was comparable to that of changes in nociceptor architecture. The long-lasting effect of a single injection of A-443654 on mechanical sensitivity (4 days) is also in line with the structural nature of this phenomenon. Third, mTORC2 activation does not affect the intrinsic excitability of sensory neurons, as A-443654 did not change passive and active membrane properties in vitro or sciatic nerve stimulation-evoked activity of DRG somata in vivo. Fourth, computational modeling demonstrated that the observed alterations in distal ending architecture are sufficient to increase neuronal excitability. Our model predicted that increases in the length of the main terminal nociceptive fiber and its branching (Figure 2) would cause a substantial rise in the AP firing rate as well as a change in timing pattern of firing. Together, our results suggest the presence of a functional link among changes in nociceptive distal endings’ structure, increased neuronal excitability, and the development of pain.

Our work shows that the overactivation of mTORC2 contributes to inflammation-induced pain and highlights the use of Rictor-ASO as a potential therapeutic treatment for this form of pain plasticity. Indeed, there is growing interest in the use of ASOs as an RNA-targeted therapy for various pathological conditions, with many clinical trials underway for neurodegenerative diseases, diabetes, cancer, and muscular dystrophies (27–29). We found that Rictor-ASO provides greater alleviation of inflammatory pain as compared with conditional deletion of Rictor in Na_v_1.8-positive nociceptors, likely as a result of Rictor downregulation in other neuronal and nonneuronal cell types. Downregulation of mTORC2 in immune cells, keratinocytes, and nociceptive Schwann cells, which all play key roles in mediating pain (30–35), may contribute to this effect.

In our study, we used *Scn10a* (Na_v_1.8^+^) *Cre* mice to target nociceptors. However, Na_v_1.8 is also expressed in myelinated A fibers (~40% of all Na_v_1.8^+^ neurons) (36), including a small population of Aβ fibers (~18%) (37). Since our study is focused on the glabrous skin epidermis, which is primarily innervated by small fibers, *Scn10a Cre* mice provide appropriate targeting of small nociceptive fibers in the epidermis.

Structural changes in primary sensory neurons have been previously described in several pathological conditions associated with pain (38). Sprouting of sensory fibers in models of cancer-induced pain (39, 40), diabetic neuropathy (41), chemotherapy-induced neuropathy (42), osteoarthritis (43), and Achilles tendinosis (44) as well as delayed growth of sympathetic postganglionic efferents after peripheral nerve injury have been well documented (45, 46). In these studies, sprouting presents as massive hyperinnervation in the affected tissue. The morphological remodeling of nociceptive peripheral terminals uncovered in our work in response to tissue inflammation is different from that in previous studies, as it is restricted to the epidermis or a superficial layer of corneal epithelium and displays as alterations in the fine morphology of the nociceptive terminal tree, resulting in a more complex distal fiber architecture. Future studies, employing high-resolution imaging and detailed morphological analysis, should establish whether such changes are also present in human chronic pain conditions.

Although we show that the effect of mTORC2 activation on structural plasticity and pain is mediated via Rac1/Cdc42 kinases, which regulate actin dynamics downstream of mTORC2, we do not directly demonstrate changes in actin in free nerve endings. Because of the ubiquitous cutaneous expression of actin in different cell types, monitoring actin dynamics in fine nerve terminals is challenging.

In summary, our work shows that peripheral inflammation induces mTORC2-mediated modulation of nociceptive distal ending architecture and provides evidence that this process contributes to the development of pain. Thus, mTORC2 represents a therapeutic target in painful conditions associated with peripheral inflammation.

## Methods

### Animals

*Rictor^loxP/loxP^* mice (47) were crossed with mice expressing Cre recombinase under the control of the *Scn10a* (Na_v_1.8) promoter (14) (provided by John N. Wood, University College London, London, United Kingdom) to generate the cKO animals *Rictor^loxP/loxP^ Scn10a^Cre^* (referred to as Rictor-cKO). Control animals used were *Scn10a^Cre^*. For imaging experiments, cKO *Rictor^loxP/loxP^ Scn10a^Cre^* mice were crossed with *TdTomato* reporter mice (Ai14, Jackson Laboratory, JAX, stock 007914) to generate *Rictor^loxP/loxP^ Scn10a^Cre^ tdTomato^loxP/loxP^*; control animals used were *Scn10a^Cre^ tdTomato^loxP/loxP^*. In order to label *Scn10a^Cre^*-positive sensory neurons for in vivo calcium imaging, *Scn10a^Cre^* mice were crossed with Rosa26-GCaMP6s mice (Ai96, Jackson Laboratory, JAX, stock 028866) or postnatal day 5 *Scn10a^Cre^* mouse pups were injected (intraplantar) with AAV9.CAG.Flex.GCaMP6s.WPRE.SV40 (Addgene, 100842-AAV9) (48). For ASO experiments, C57BL/6 mice (Jackson Laboratory, JAX, stock 000664) were used. All experiments were conducted on 8- to 12-week-old male and female mice on the C57BL/6 background. To assess for the presence of main effects of sex or interactions of sex with experimental manipulations, key data sets (Figure 1, F, G, I, and J, Supplemental Figure 2, A–D, and Supplemental Figure 3, C–F) were analyzed using sex as a factor. In no cases were any main effects of sex or interactions observed, so data were collapsed for all reported analyses. Food and water were available ad libitum. Mice were kept on a 12-hour light/12-hour dark cycle (lights on at 7:00 am). Behavioral experiments were conducted during the light phase of the cycle (0900–1700 hours). In the absence of expected effect sizes, sample sizes were determined based on previous behavioral, molecular, and electrophysiological data published by our laboratories. In all experiments, animals were randomly assigned to treatment groups. Experimenters were blinded to genotype, drug treatment, and pain models during data acquisition and analysis.

### Surgically induced animal models of pain

All animals were 8 weeks of age when undergoing surgical procedures. Neuropathic pain was induced by performing SNI (49) and CCI (50) procedures. For the SNI, the lateral surface of the thigh skin was shaved and incised, the left sciatic nerve was isolated, and the tibial and common peroneal branches were ligated using 7-0 silicone-coated silk (Covidien, S-768K); a 3 mm portion of each branch was sectioned and removed distal to the ligation point. For the CCI, the sciatic nerve was ligated 3 times using 7-0 silicone-coated silk (all under 2% isoflurane). The muscle and skin were closed with 6-0 VICRYL Suture (Ethicon, J489G).

### Behavioral testing

For the von Frey assay, mice were habituated in individual transparent Plexiglas cubicles (5 × 8.5 × 6 cm) set above a perforated steel floor for 1 hour prior to testing. Nylon monofilaments were firmly applied to the plantar surface of each hind paw for 3 seconds. The up-down method of Dixon was used to estimate the 50% withdrawal threshold (average of 2 measurements separated by at least 30 minutes).

For the radiant heat paw-withdrawal assay, mice were habituated in individual transparent Plexiglas cubicles (5 × 8.5 × 6 cm) set above a transparent glass floor for 1 hour prior to testing. A focused beam of high-intensity light was aimed at the plantar surface of the hind paw. The intensity was set at 20% of the maximum (IITC model 390) with a cut-off value of 40 seconds. Latency to hind paw withdrawal was measured to the nearest 0.01 seconds. Both hind paws were measured twice on 2 separate occasions separated by at least 30 minutes.

For the capsaicin eye-wipe test, animals were habituated in individual transparent Plexiglas cubicles (5 × 8.5 × 6 cm) set on a solid wooden surface for 1 hour prior to testing. The numbers of eye wipes and blinks were determined after video recording for 5 minutes. When performing UV irradiation (VL-6.C lamp, Montreal-Biotech Inc.), the protocol of Acosta et al. (51) was followed with modifications for mice (21). Briefly, animals were anesthetized and situated 17 cm from the UV lamp. Both eyes were irradiated for 22 minutes with UV-C radiation (wavelength of 254 nm), delivering a radiation intensity of 450 mJ/cm^2^. Twenty-four hours following UV irradiation, animals were habituated for 1 hour prior to administration of 10 μM drops of 1 μM capsaicin into the left eyes. The numbers of eye wipes and blinks were determined after video recording for 5 minutes.

For the tail-clip assay, a binder clip exerting a force of 155 grams (g) or 248 g was applied 1 cm from the base of the tail. Animals were placed in an enclosure (15 × 25 cm), and the latency to attack the clip was measured. The cutoff for responses was set at 60 seconds.

For the hot-plate assay, we used a hot-plate apparatus (Columbus Instruments) set at either 50°C or 53°C. Animals were placed on the hot plate enclosed within an open Plexiglas cylindrical tube, and latency to demonstrating any nocifensive responses — jumping, hind paw licking, or rapid fluttering/shaking of the hind paw —was measured. The cutoff for response was set at 90 seconds and 60 seconds, respectively.

For the tail-flick assay, animals were restrained in a cloth restrainer, and the tail was placed into a water bath of either 47°C or 49°C. The latency to the animal flicking its tail was measured. The cutoff for response was set at 30 seconds and 15 seconds, respectively.

MGS was performed as previously described (52). Mice were placed in custom-made Plexiglas cubicles (5.3 × 8.5 × 3.6 cm) on a perforated metal floor and were habituated for 30 minutes prior to testing. Mice were recorded for 1 hour with a digital video camera (Sony Digital Camcorder HDR-PJ430V). A total of 20 head-shot photos were used to define a mean score for each animal, in which 1 photo was taken at 3-minute intervals within the 1 hour recording. Photos of sleeping mice were discarded as well as photos of mice expressing grooming or scratching behavior. Coders were subsequently blinded, and photos were randomized, then scored using the following criteria: the intensity rated as value of 0, 1, or 2 for each of the 5 action units (AUs). This included orbital tightening, nose bulge, cheek bulge, ear position,and whisker change. In every case, 0 indicated the AU was not present, 1 indicated moderate visibility of the AU, and 2 indicated severe presentation of the AU. An MGS score for each photo was calculated by averaging intensity ratings for each AU.

### Inflammation-induced animal models of pain

In CFA, 1:1 complete and incomplete Freund’s adjuvants were mixed (Sigma-Aldrich) to produce an emulsion, 20 μL of which was delivered under the skin of the left hind paw (intraplantar). γ-Carrageenan (Sigma-Aldrich) was prepared at 0.5% in double-distilled water (ddH_2_O), and 20 μL was injected into the left hind paw (intraplantar). Capsaicin (Sigma-Aldrich) was prepared at 1 μM in 0.1% ethanol saline solution consisting of the following: 145 mM NaCl, 5 mM KCl, 2 mM CaCl_2_, 1 mM MgCl_2_, 10 mM glucose, 10 mM HEPES; 10 μL was applied onto the eye.

### Drugs

The mTORC2 activator A-443654 (MCE) was made up to a 40 mM stock in DMSO, and 20 μL of 8 mM dilution made in 4% PEG, 4% DMSO in 0.9% saline was injected beneath the skin of the left hind paw (intraplantar). The Rac1 inhibitor NSC23766 (EMD Millipore) was prepared in a 50 mM stock solution in distilled water, and 10 μL of 10 mM dilution in 0.9% saline was injected beneath the skin of the left hind paw. The Cdc42 inhibitor ML141 (EMD Millipore) was made up to a 100 μM stock in DMSO, and 10 μL of 50 μM dilution in 0.9% saline was injected beneath the skin of the left hind paw. The rapalog CCI-779 (Sigma-Aldrich) was made up to a 50 mg/mL stock in DMSO and was then diluted to a dose of 25 mg/kg in 5% PEG, 5% Tween-80 in saline.

### Western blotting

Samples were homogenized in ice-cold lysis buffer consisting of 200 mM HEPES, 50 mM NaCl, 10% glycerol, 1% Triton X-100, 1 mM EDTA, 50 mM NaF, 2 mM Na_3_VO_4_, 25 mM β-glycerophosphate, and EDTA-free complete ULTRA tablet. A total of 30 μg of protein per sample was resolved on SDS-PAGE (8%), transferred onto nitrocellulose membranes, and imaged using a ChemiDoc Imaging System (Bio-Rad). Primary antibodies for Western blotting were Rictor (catalog 2114S), p-Akt (Ser473; catalog 4060S), t-Akt (catalog 4691S), and GAPDH (catalog 2118S) raised in rabbit (Cell Signaling Technology). Secondary antibody for Western blotting was anti-rabbit HRP (catalog RPN4301, GE Healthcare).

### Immunohistochemistry

Animals were perfused via the left cardiac ventricle with 10 mL of 0.1% NaNO_2_ perfusion buffer (64 g NaCl, 2 g KCl, 4 g NaHCO_3_, 400 mL 0.2M phosphate buffer [PB] in 8 L of ddH_2_O), followed by 100 mL of 4% paraformaldehyde in 0.1M PB. The lumbar portion of the spinal cord, glabrous hind paw skin, DRGs, and cornea were extracted and post-fixed in the same fixative solution at 4°C. Skin, DRG, and cornea tissue were transferred 24 hours later to a cryoprotectant solution consisting of 30% sucrose in 0.1M PB, embedded in OCT (Thermo Fisher Scientific), sectioned at 25, 14, and 25 μm, respectively, using a Leica cryostat, and collected directly onto gelatin-subbed histological slides. Spinal cord was sectioned at 30 μm in cross sections using a Leica vibratome and collected as free-floating in PBS into 24-well plates. Paw skin and cornea sections were washed with PBS to remove residual OCT and coverslipped using Aqua Poly/Mount (Polysciences Inc.). Spinal cord tissue was directly coverslipped using ProLong Gold Anti-Fade (Thermo Fisher Scientific) and mounted onto histological slides.

To detect peptidergic and nonpeptidergic C-fiber nociceptors in the DRG and glabrous skin, OCT was removed from sections with three 10-minute washes using 0.2% Triton X-100 in PBS (PBS-T) and incubated for 24 hours at 4°C in rabbit anti-CGRP (1:500; catalog C8198, Sigma-Aldrich) and IB4 lectin conjugated to Alexa Fluor 488 (1:500, catalog I21411, Thermo Fisher Scientific) in PBS-T. The next day, after several washes with PBS-T, sections were incubated in goat anti-rabbit secondary antibody (Alexa Fluor 647, 1:500, catalog A32733, Thermo Fisher Scientific) in PBS-T for 2 hours at room temperature. Sections were then washed with PBS-T, incubated with fluorescent Nissl (1:20, Invitrogen) in PBS-T for 20 minutes, washed again using PBS-T for 10 minutes, mounted onto gelatin-subbed slides, and coverslipped using Aqua Poly/Mount (Polysciences Inc.). To detect myelinated A-fiber cells, DRGs were also stained with mouse anti-NF200 (1:500, catalog N5389, Sigma-Aldrich) and goat anti-mouse secondary antibody (Alexa Fluor 568, 1:500, catalog A11004, Thermo Fisher Scientific).

To measure p-Akt, DRGs were incubated for 24 hours at 4°C in rabbit anti–p-Akt (Ser473) (1:500, catalog 4060S, Cell Signaling Technology) and incubated in goat anti-rabbit secondary antibody for 2 hours at room temperature (1:500, Alexa Fluor 488, catalog A11034, Thermo Fisher Scientific).

#### Confocal imaging.

Paw skin, DRGs, cornea, and spinal cord sections were imaged using an LSM 880 confocal microscope with AiryScan (Zeiss). The paw skin and cornea were imaged with the ×63 oil immersion objective using only the 568 nm channel. *Z*-stacking was conducted, with a step interval of 0.5 μm. An area of 8 tiles (135 × 260 μm) was imaged for all sections. DRGs were imaged with the 10× objective using 4 channels. *Z*-stacking was conducted with a step interval of 1 μm. An area of 12 tiles (1422 × 1896 μm) was imaged for all sections. The spinal cord was imaged with a ×20 objective using the 568 nm channel. *Z*-stacking was conducted with a step interval of 1 μm. An area of 15 tiles (711 × 1185.5 μm) was imaged for all sections. A total of 8 to 10 sections were imaged per animal.

For the high-resolution analysis of nociceptive fiber architecture in the glabrous paw skin and cornea, imaging using the AiryScan mode was conducted using Zeiss ×63/1.40 oil DIC f/ELYRA objective and the AiryScan super-resolution (SR) module with 32-channel hexagonal array GaAsP detector for LSM (Zeiss) using 651 nm lasers. Stacks of 20 to 30 optical sections (170 nm step) were acquired, and AiryScan SR image stacks were reconstructed using ZEN Black software (Zeiss). Images were analyzed using ZEN Blue software (Zeiss) and ImageJ (NIH).

#### Image analysis quantification.

The quantification of sensory fibers in the paw skin and cornea was performed with ImageJ software. Prior to any type of analysis, a consistent live frame area was measured (123.94 μm × 72.33 μm) and placed in an area where fibers were most dense. The number of main fibers was determined by counting tdTomato-positive fibers in the epidermis of glabrous paw skin. Main fibers were distinguished from the axons in the upper dermis on the basis of growth, relative width, and location. In addition to main fibers being counted, their length was measured with the free-hand tool in ImageJ software. Smaller fibers emerging from the main fiber were classified as branches. Quantification of these branches was performed using length measurement, similar to main fiber measurement, beginning at the base of its sprout from the main fiber until it terminated. The distance from the junction where these branch structures protrude to the axon where main fibers innervate was measured.

DRGs were quantified using the cell counter app in ImageJ. Colocalization of anti-CGRP, IB4, or anti-NF200 with fluorescent Nissl indicated a positive cell. Proportions of cell populations were acquired by taking the ratio of positive cells for 1 of the 3 sensory fiber markers to the total number of Nissl-stained cells. A similar approach was used to quantify p-Akt, where the ratio of colocalized cells for p-Akt and Na_v_1.8 tdTomato to all Na_v_1.8 tdTomato cells was calculated.

The quantification of TdTomato-labeled primary afferents in the spinal cord was done using ImageJ. The intensity was analyzed by measuring the mean gray value using a consistent live frame area (123.94 μm × 72.33 μm) for all sections. The width of the primary afferents was measured by drawing a perpendicular line from one side of the Na_v_1.8 tdTomato-labeled afferents to the other in ImageJ.

### Calcium imaging in vivo

Adult *Scn10a^Cre^*;GCaMP6s mice were deeply anesthetized with 100 mg/kg ketamine, 15 mg/kg xylazine, and 2.5 mg/kg acepromazine (A7111, Sigma-Aldrich). L4 DRG was exposed with laminectomy. The sciatic nerve on one side was exposed, and a pair of hook electrodes were placed under the sciatic nerve to deliver electrical stimulation. The spinal columns flanking the surgical exposure were clamped with 2 clamps of a custom spinal stabilization device to fix the animal, and 3% agar solution was used to make a pool for holding Ringer solution (126 mM NaCl, 2.5 mM KCl, 2 mM CaCl2, 2 mM MgCl2, 10 mM d-Glucose, 10 mM HEPES, pH = 7.0). The animal’s body temperature was maintained at 37°C with a heating pad during the surgery and throughout the imaging experiment. Warmed Ringer solution was dropped on the exposed spinal cord and DRGs and repeatedly changed during the imaging experiment.

Animals with the whole spinal stabilization device were fixed under a homemade video-rate 2-photon microscope. A tunable InSight X3 Femto-Second laser (Spectra-Physics) was set to 940 nm for GCaMP6s imaging. Images were acquired at 32 Hz with an Olympus water-immersion ×40 objective at a resolution of 0.375 μm/pixel (48).

For electrical stimulation, 5 pulses of 5 mA or 9 mA electrical current with 1 ms duration at 5 Hz were delivered to the exposed sciatic nerve using a digital stimulator (PG4000A, Cygnus Technologies) and a stimulus isolation unit (A365, World Precision Instruments). The in-house acquisition software of the 2-photon microscope triggered the digital stimulator using a data acquisition card (LabJack, U3-HV).

#### Image processing and analysis.

The recorded images were processed and analyzed as previously reported (48). Briefly, the RAW image sequences were converted into TIFF format in ImageJ. Then rigid body translation alignment based on 2D cross-correlation was performed with a custom-built MATLAB (MathWorks) function to correct for movement when needed. A rectangular region of interest (ROI) in a region absent of any visible neurons was drawn as background ROI. The average pixel value inside the background ROI for each frame was subtracted from every pixel in the corresponding frame. Then rectangular ROIs were placed manually in the cytoplasm of visible neurons. The average fluorescence intensity of a given ROI, *F_t_*, was measured by averaging pixel values inside the ROI. Calcium traces were calculated as follows: Δ*F/F_0_* = (*F_t_* – *F_0_*)/*F_0_*, where *F_0_* is the fluorescence value at baseline, which was measured as the average of the first 2 seconds of *F_t_*. To avoid aberrant amplification due to small *F_0_* values in some neurons (e.g., low basal fluorescence) when it was less than 1, *F_0_* in the denominator, but not in the numerator, was set to 1. Processing was performed using custom functions written in MATLAB.

An integrated interface within a custom tool written in Spike2 (CED) was used to automatically detect and measure positive responses. Raw Ca^2+^ traces were first smoothed with a 1-second temporal window. Baseline was selected from a period between 1 second after the beginning of the recording and 1 second before the stimulus onset (usually 8 seconds in duration). Then, *F_b_* and *F_b-max_* were calculated as average and maximum Δ*F/F_0_* values during baseline, respectively. A response was considered positive when the peak of the Ca^2+^ trace during stimulation was above *F_b_* + (*F_b-max_* – *F_b_*) × *x*, where *x* was a value between 2 and 3, depending on baseline stability, to provide the most reliable detection. Given the relatively slow decay of GCaMP6s, responses with very brief duration (<0.5 seconds) were excluded. While the detection algorithm was found to be highly reliable, all traces were also visually inspected to ensure no false positives were included and no false negatives missed. Peak amplitude was measured for all responses using automated algorithms. For each neuron, each parameter was measured for each of the positive responses obtained over multiple trials and then averaged.

### Patch-clamp electrophysiology

#### DRG cell culture preparation.

DRGs were extracted from 8- to 12-week-old *Scn10a^Cre^ tdTomato* mice. Animals were injected with mTORC2 activator A-443654 (MCE, 8 mM, 2.5 mg/kg, dissolved in 4% PEG, 4% DMSO, in 0.9% saline, i.p.) or control vehicle (saline). Briefly, DRG neurons were removed and incubated in HBSS containing dispase (1.37 mg/mL, Gibco, Thermo Fisher Scientific) and collagenase II (1.08 mg/mL, Gibco, Thermo Fisher Scientific) for 30 minutes at 37 °C. After 2 washes in complete medium (Ham’s F-12 Nutrient Mixture, 10% [vol/vol] FBS, 2 mM l-glutamine, and 1% penicillin-streptomycin), DRGs were mechanically dissociated using 3 fire-polished Pasteur pipettes with sequentially decreasing diameters. The cells were concentrated by centrifugation (193*g* for 2 minutes), resuspended in culture medium, and plated on the glass bottom of a 35 mm dish precoated with a mixture of 100 μg/mL poly-d-lysine and 10 μg/mL laminin in HBSS. For in vitro application, 1 μM of A-443654 or vehicle was applied to cultures for 10 minutes prior to recordings.

#### Current clamp recordings.

Recordings were performed from Na_v_1.8^+^ and less than 25 μm diameter dissociated DRG neurons up to 24 hours after plating. Whole-cell membrane voltages were recorded using a Multiclamp 700B amplifier (Molecular Devices) at room temperature (24 ± 2°C). Data were sampled at 20 kHz and low pass filtered at 500 Hz. Patch pipettes (4–8 MΩ) were pulled from borosilicate glass capillaries and fire polished. The intracellular solution contained the following: 130 mM K-gluconate, 10 mM HEPES, 5 mM EGTA, 3 mM Mg-ATP, and 0.4 mM GTP (pH 7.3). The extracellular solution contained the following: 150 mM NaCl, 5 mM KCl, 1 mM MgCl_2_, 2 mM CaCl_2_ 10 mM HEPES, and 10 mM d-glucose. The pH and osmolarity were adjusted to 7.4 and 300, respectively. Command current protocols were generated with a Digidata 1550B A/D interface (Molecular Devices). Data were digitized using pCLAMP software, version 10.3 (Molecular Devices). Data analysis was performed using Clampfit software, version 10.7.

Neurons were recorded under current clamp at RMP. Cells with RMP with a depolarization of less than –40 mV or those in which the access resistance changed more than 25% from its initial value during the recordings were excluded. The membrane potential was not corrected for liquid junction potential (+5.6 mV). A series of depolarizing square current injections (500 ms with a 2-second interval and 50 pA increments) were used to assess AP parameters measured from the first AP evoked. Input resistance and the sag were measured by separate hyperpolarizing square pulses at –20 pA and –80 pA, respectively. Number of spikes was measured by injecting ramp current from baseline to 600 pA over 2 seconds to mimic slow depolarization.

### DRG cell culture

#### Immunostaining.

DRG cell cultures were treated with vehicle saline or A-443654 (1 μM) for 10 minutes. Culture media was removed, and cells were fixed with 4% paraformaldehyde in PBS with 5 mM MgCl_2_, 4% sucrose, and 0.1 mM CaCl_2_ (PBSM). Cultures were then quenched with PBSM with 0.1M glycine for 10 minutes followed by 2 washes with PBS. Cells were permeabilized with PBS supplemented with 0.1% Triton X-100 for 10 minutes, washed with PBS, and prehybridized for 1 hour with PBS with 0.05% Tween and 10% NGS. To measure A-443654 activity, primary antibody rabbit anti–p-AKT (Ser473) (1:500, catalog 4060S; Cell Signaling Technology) was applied at 4°C for 24 hours. The next day, cells were washed twice with PBS with 0.05% Tween and 10% NGS, incubated with goat anti-rabbit secondary antibody for 1 hour at room temperature (1:500, catalog A11034, Alexa Fluor 488; Thermo Fisher Scientific), washed twice again, and coverslipped using ProLong Gold.

#### Confocal imaging and analysis.

Cells were imaged using an LSM 880 confocal microscope with Airyscan (Zeiss) with a ×63 oil immersion objective using the 488 and 568 nm channels. *Z*-stacking was conducted, with a step interval of 0.2 μm. The quantification of p-Akt in tdTomato-positive cells was performed with ImageJ software. A consistent area was placed in the same location for all cells, and the integrated density value was measured (background subtracted). Eight to ten cells were measured per animal, with a total of *n =* 3 animals per condition.

### Computational modeling

Simulations were based on our previously developed and published biophysically realistic multicompartment model of an unmyelinated axon (22, 53). Briefly, simulations were performed in a NEURON 7.5 simulation environment. The nociceptor morphology consisted of a 25 μm diameter soma-like compartment attached to a stem axon expanding to peripheral and central axons that joined at a T-junction bifurcation site. The branches were of a 0.25 μm diameter. Terminal length morphology was extended by length and expanded by number with respect to this study’s findings (see Figure 5).

#### Passive membrane properties.

The intrinsic membrane properties were also as stated in Barkai et al. (22): passive membrane resistance of 10,000 Ω cm^−2^ was set for all compartments apart from the terminal branch, and axial resistance (Ra) in all compartments, apart from the terminal branch, was 150 Ω cm, which had a 4-fold somatic membrane resistance (54). All compartments had a membrane capacitance of 1 μF cm^−2^. The passive reversal potential (*E_Pas_*) was set to −60 mV.

#### Active conductances.

The model consisted of TTX-sensitive sodium current (*I_NattxS_*), TTX-sensitive persistent sodium current (*I_NaP_*), Na_v_1.9 TTX-resistant sodium channels (*I_Nav1.9_*), and Na_v_1.8 TTX-resistant sodium channels (*I_Nav1.8_*). All channel parameters of the sodium currents were adapted from Herzog et al. (55) and Baker (56). Three types of potassium channels included the following: (a) the delayed rectifier channel (*I_KDR_*), adapted from Herzog et al. (55); (b) an A-type potassium channel (*I_KA_*), adapted from Miyasho et al. (57), whose activation and inactivation gates were shifted by 20 mV in the hyperpolarized direction to closely resemble the kinetics of DRG neurons (58), and (c) the Kv7/M channels adapted from Shah et al. (59) and their activation curve parameters, tuned as in Barkai et al. (53). The h-current (*I*_h_) was also included and taken from Shah et al. (59), and the slope factor was tuned according to Komagiri and Kitamura (60). The T-type and L-type channels represent the low voltage–activated (LVA) (*I_CaL_*) and high voltage–activated (HVA) currents (*I_CaT_*). Specific channel conductances (*g*) were as previously described (22): *g_Nav1.8_* = 0.02 *S*/cm^2^, *g_Nav1.9_* = 0.00064 *S*/cm^2^, *g_NaTTXS_* = 0.0017 *S*/cm^2^, *g_NaP_* = 0.00005 *S*/cm^2^, *g_KDR_* = 0.00083 *S*/cm^2^, *g_KA_* = 0.0015 *S*/cm^2^, *g_Kv7/M_* = 0.00034 *S*/cm^2^, *g_H_* = 0.00033 *S*/cm^2^, *g_CaL_* = 0.003 *S*/cm^2^, *g_CaT_* = 0.001 *S*/cm^2^.

The reversal potentials for sodium (*E_Na_*), potassium (*E_K_*), and h-current (*E_H_*) were set to +60mV, −85mV, and −20 mV, respectively. Apart from sodium conductances, which were unevenly distributed between Na_v_-less and conductive compartments, all other conductances were evenly distributed in all compartments.

The terminating branches were divided into 2 sections separated by the SIZ, a 25 μm long compartment completely absent of sodium conductances (Na[v]less compartment) and a 50 μm long “propagation” compartment connecting between the Na(v)less compartment and the junction with the rest of the terminal branch (21, 22). The inflammation-induced de novo branches were considered as Na(v)less compartments. The propagation compartments and de novo branches’ lengths were changed (by percentages), as stated in Figure 5.

Since Nav1.9 TTX-resistant sodium channels (*I_Nav1.9_*) are expressed preferentially by nonpeptidergic neurons (61), to simulate peptidergic-like nociceptive neurons, we removed Na_v_1.9 TTX-resistant sodium channel (*I_Nav1.9_*) conductance from the model, leaving all other active and passive parameters intact. The architecture of the terminal fibers was also modified (the basal terminal fiber length was shortened and the number of branches decreased) with respect to our (Figure 2 and Supplemental Figure 5) and others’ (17) findings.

#### Nerve-ending stimulation.

A capsaicin-like stimulation was injected to all nerve endings. A capsaicin-like current was introduced into a single simplified voltage-clamp point process with fast exponential activation and slow exponential inactivation mimicking the experimental kinetics of puff-applied 1 μM capsaicin-induced current and sufficient to induce AP firing when applied onto the acutely dissociated DRG neuron (62). A similar current was used previously (21, 22). To simulate the opening of transducer channels during the stimulation of capsaicin, we introduced transducer channel conductance into the stimulated nerve ending as described previously (22). The conductance to this channel was exponentially distributed and the exponential decay constant (γ) was calculated to fit the diffusion of capsaicin and hence the change in its concentration as a function of distance from the pipette tip as calculated previously (21).

The final transducer distribution equation introduced into the model was as follows: *g_Trans_* = *ḡ_Trans_* × *e^–x/y^*, where *x* is the distance from the nerve ending and the fixed parameters were *ḡ_Trans_* = 0.0025 *S*/cm^2^ and *y* = 7.04 μm.

Free-ending terminal branch axial resistance (Ra) was increased to ×15 of the distal axon Ra to simulate the localization of intracellular organelles in the free-ending terminal branches (21, 22, 63, 64).

A capsaicin-like induced current was introduced into a single simplified voltage clamp point process with a fast exponential activation and slow exponential inactivation mimicking the experimental kinetics of puff-applied 1 μM capsaicin-induced current as used previously (22):

(Equation 1) 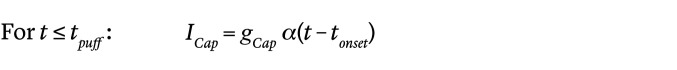


(Equations 2, 3, and 4) 
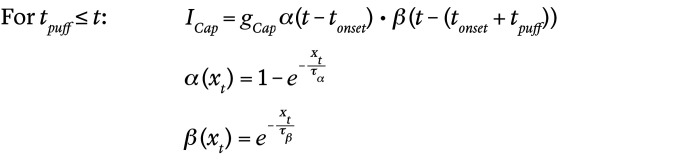


where *g_Cap_* is the maximal conductance, *α*(*x_t_*) and β(*x_t_*) are the activation and inactivation functions with τ_α_ and τ_β_ as the activation and inactivation time constants, respectively. τ_α_ and τ_β_ were applied with the following values: *τ_α_* = 1 × 10^6^ ms and *τ_β_* = 6500 ms; *t_onset_* and *t_puff_* are the times at which the puff application simulation begins and the length of application, respectively. The recordings were performed by positioning a NEURON “point-process” electrode at the terminal end of the central axon (22).

#### Data and software availability.

The model files are available in the ModelDB repository (accession 266850).

### Antisense oligonucleotide synthesis and administration

Rictor-ASO (GTTCACCCTATACATTACCA) targeting mouse *Rictor* mRNA and control-ASO, which does not target any sequence in the mouse genome (CCTATAGGACTATCCAGGAA), were developed and synthesized by Ionis Pharmaceuticals. Each ASO consists of 5 nucleotides on the 5′ and 3′ ends of the ASO with a 2′-*O*-methoxyethyl (MOE) modification and a central 10-base DNA “gap.” Rictor-ASO was confirmed to bind with 100% complementarity to mouse *Rictor* mRNA and does not bind to any other mRNA with full complementarity within the mouse transcriptome. Lyophilized Rictor and control ASO were diluted in Dulbecco’s phosphate-buffered saline to a concentration of 100 mg/ml and were delivered s.c. at a single dose of 100 mg/kg.

### Statistics

All data are reported as mean ± SEM and were analyzed using GraphPad Prism 9. A *P* value of less than 0.05 was considered significant. Unpaired Student’s *t* test (2 tailed), 1-way and 2-way ANOVA, and repeated measures ANOVA were used, where appropriate, to analyze data.

### Study approval

Housing and all experimental procedures on mice complied with the guidelines of the Canadian Council on Animal Care and the International Association for the Study of Pain and were approved by McGill University’s Downtown Animal Care Committee and Laval University’s Animal Care Committee.

## Author contributions

CW, ARDS, AMB, and AK conceived and designed the study and supervised experiments. CW performed surgeries, behavioral experiments, immunohistochemistry/imaging, and Western blotting experiments. OB conducted computational modeling. FW conducted in vivo DRG calcium imaging. CTP and SL conducted patch-clamp recordings. WC scored MGS behavior. KCL assisted with SNI surgeries, and ST measured SNI behavior. ML assisted with image analysis. NY, MH, ACC, MPK, JSM, PS, and YDK assisted with study design and interpretation of results. All authors discussed the results and provided input on the manuscript. CW, AMB, and AK wrote the manuscript.

## Supplementary Material

Supplemental data

## Version 1 05/17/2022

In-Press Preview

## Version 2 08/01/2022

Electronic publication
