# Supplementary material for: mTORC2 mediates structural plasticity in distal nociceptive endings that contributes to pain hypersensitivity following inflammation
Source: J Clin Invest. 2022 Aug 1;132(15):e152635. doi: 10.1172/JCI152635 (PMC9337825; doi:10.1172/JCI152635)
Supplement: Supplemental data [file jci-132-152635-s127.pdf]

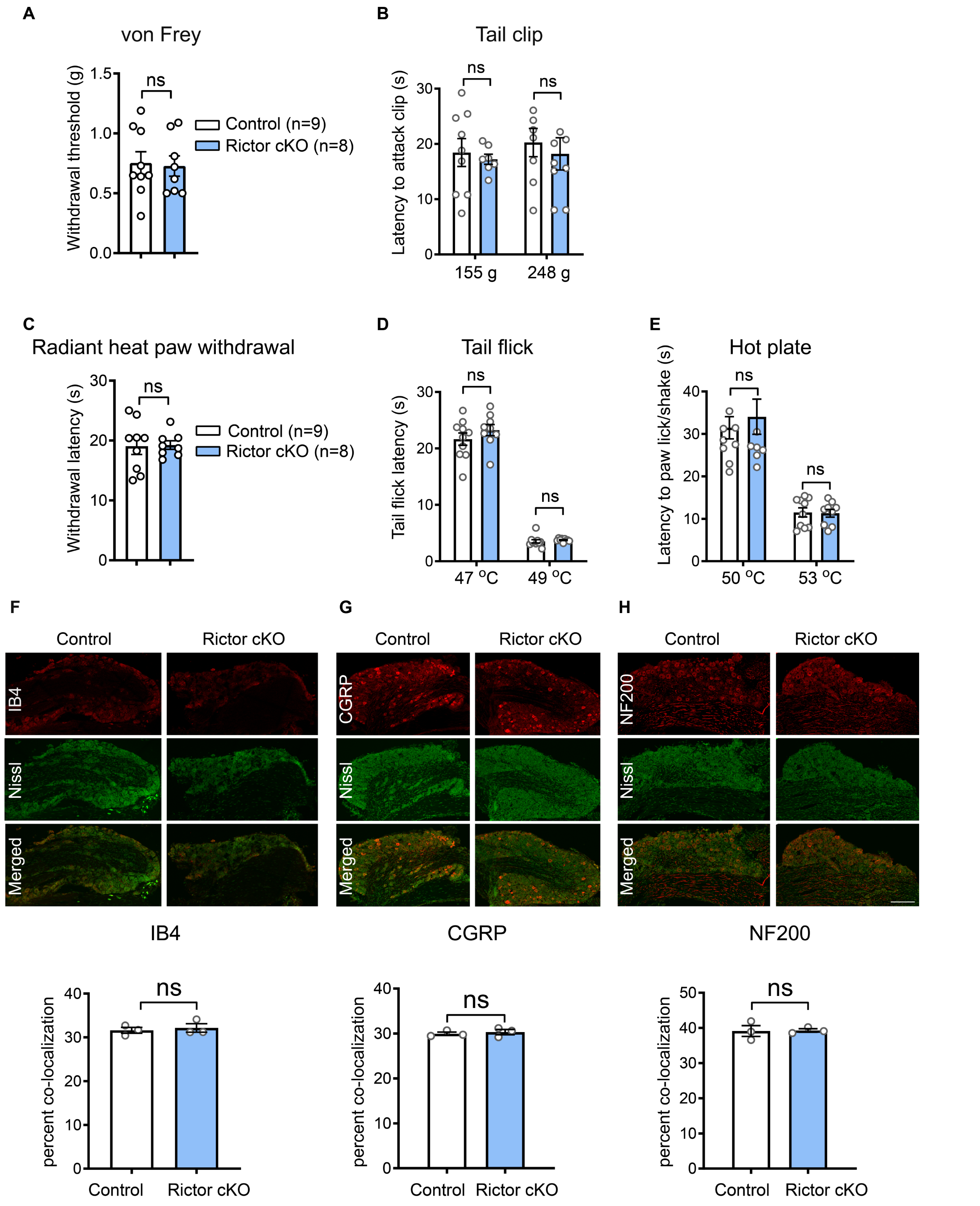


**Supplemental Figure 1. Conditional deletion of Rictor in nociceptors does not change baseline mechanical and thermal sensitivity.** No differences between Control and Rictor cKO mice in von Frey

(**A**), tail clip (**B**), radiant heat withdrawal (**C**), tail flick (**D**) and hot plate (**E**) assays (n = 8-12 mice per group). Representative images and quantification show no differences in the proportion of IB4-positive (**F**), CGRP-positive (**G**) and NF200-positive (**H**) neurons in DRG of Control and Rictor cKO mice. Student's t-test. All data are presented as mean ± s.e.m. ns, not significant. Scale bar, 200 μm.

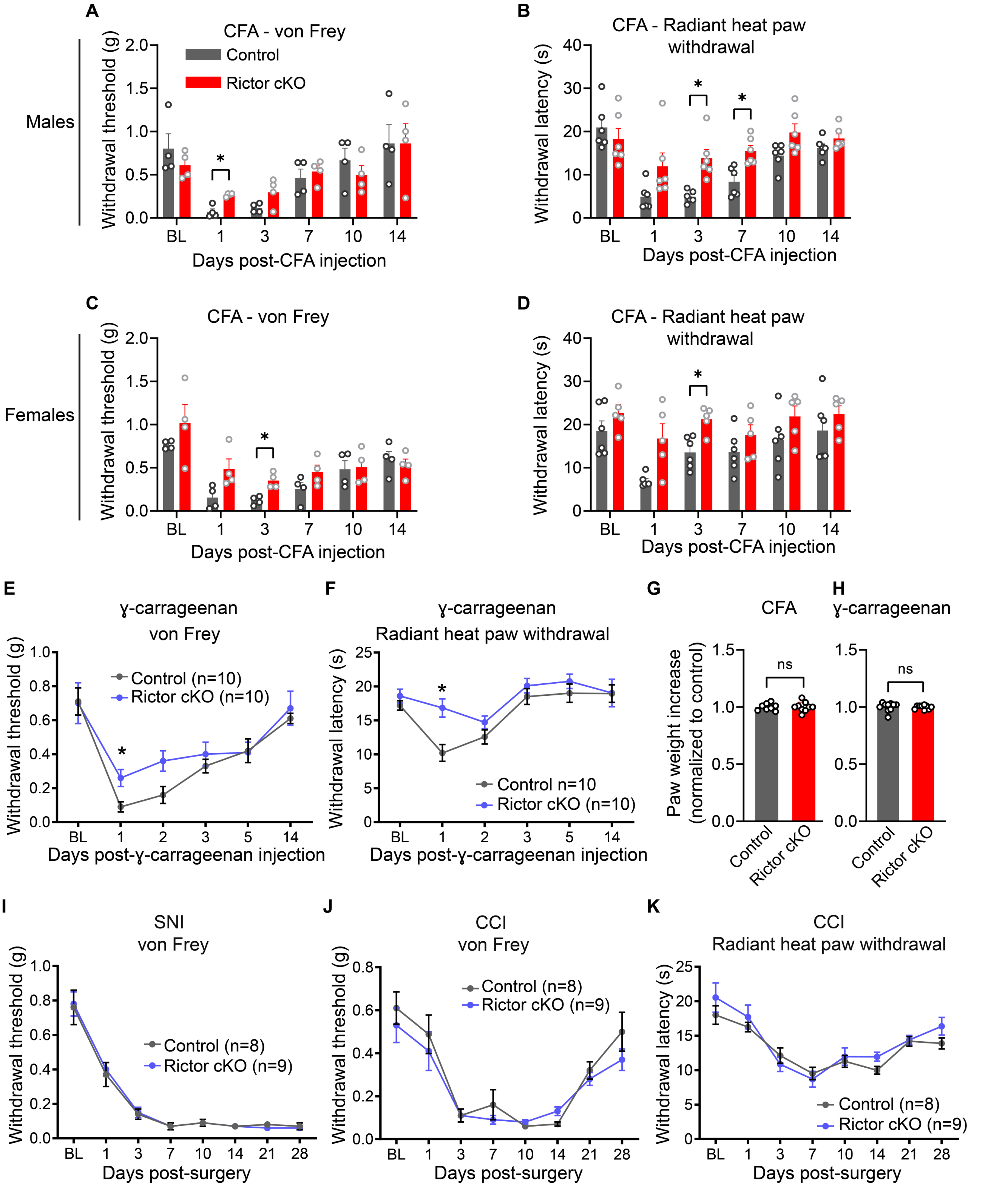


**Supplemental Figure 2. mTORC2 in nociceptors is not involved in mediating neuropathic pain.** Ablation of Rictor in nociceptors reduces intraplantar CFA-induced mechanical and thermal hypersensitivity in males and females (von Frey, **A** and **C**; radiant heat paw withdrawal, **B** and **D)**.

Intraplantar γ-carrageenan injection induces lower mechanical (**E**) and thermal (**F**) hypersensitivity in Rictor cKO mice. No differences were found in the level of inflammation in Control and Rictor cKO animals 1 day after intraplantar injection of CFA (**G**) and γ-carrageenan (**H**) (Student's t-test). Control and Rictor cKO mice show no differences in the development of mechanical hypersensitivity (von Frey) in the model of peripheral nerve injury, spared nerve injury (SNI, **I**) and no differences in mechanical (**J**) and thermal (**K**) hypersensitivity in chronic constriction injury (CCI). Statistics are based on two-way ANOVA followed by Bonferroni's post-hoc comparison (for **A-F**, **I-K**). All data are presented as mean ± s.e.m. *$p < 0.05$, ns, not significant.

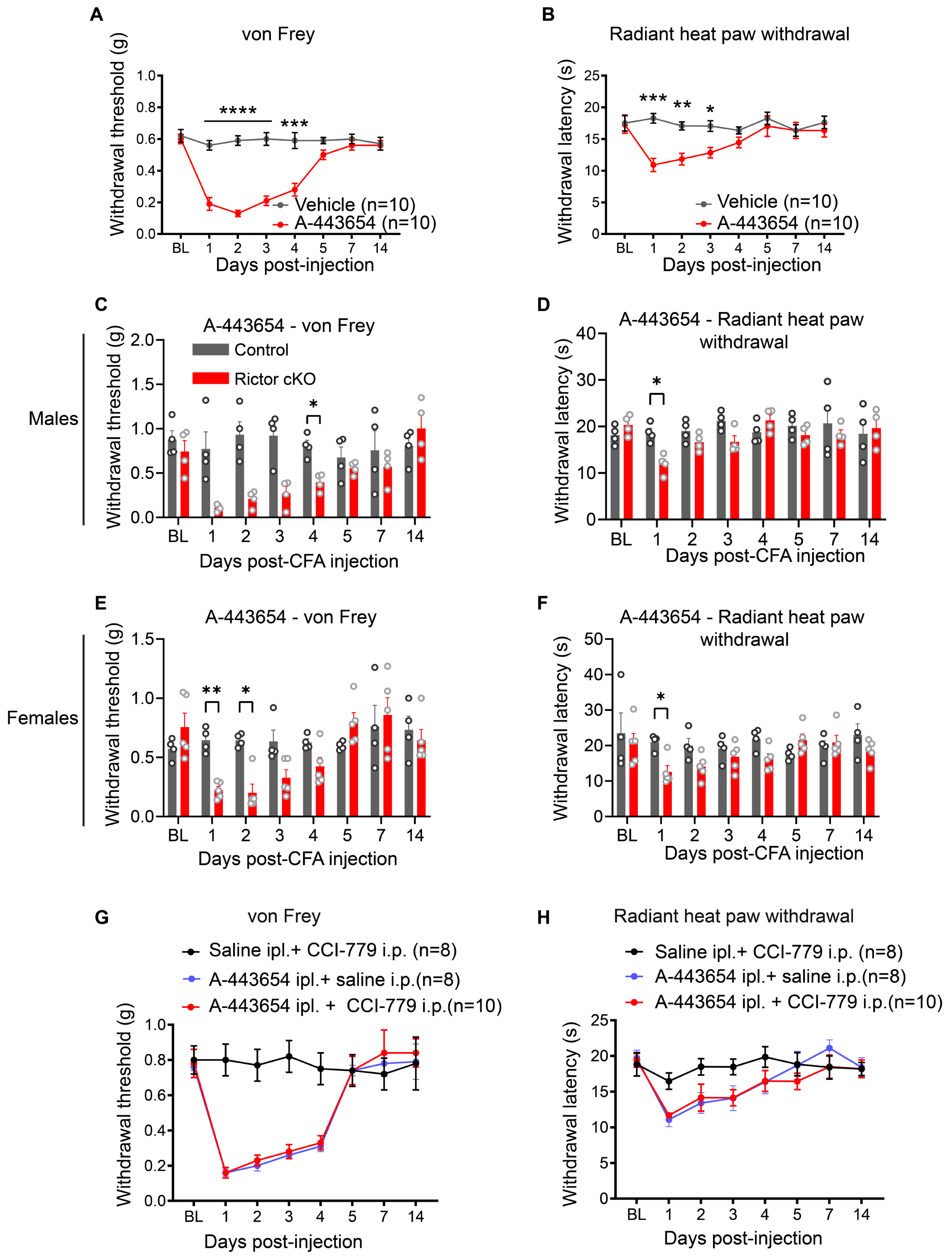


**Supplemental Figure 3. Activation of mTORC2 promotes pain hypersensitivity.** Intraperitoneal injection of the mTORC2 small molecule activator A-443654 **(**2.5 mg/kg) induced mechanical (**A**) and

thermal (**B**) hypersensitivity. Intraplantar injection of A-443654 caused mechanical and thermal hypersensitivity in male and female mice (von Frey, **C** and **E**; radiant heat paw withdrawal, **D** and **F**). Statistics are based on two-way ANOVA followed by Bonferroni's post-hoc comparison (for **A-F**). Inhibition of mTORC1 with CCI-779 (25 mg/kg i.p., 15 mins prior to A-443654, 20 µL, 8 mM, ipl.) does not prevent the development of A-443654-induced mechanical (**G**, von Frey, Vehicle + CCI-779 versus A-443654 + CCI-779 Day 1 q(7.536) = 9.673, $p = 0.0004$, Day 2 q(8.340) = 7.705, $p = 0.0014$, Day 3 q(9.339) = 7.957, $p = 0.0007$, Day 4 q(9.363) = 6.238, $p = 0.004$; Vehicle + CCI-779 versus A-443654 + Vehicle Day 1 q(8.049) = 9.427, $p = 0.0004$, Day 2 q(8.135) = 8.239, $p = 0.0009$, Day 3 q(7.804) = 8.664, $p = 0.0008$, Day 4 q(8.468) = 6.754, $p = 0.003$; A-443654 + CCI-779 versus A-443654 + Vehicle, p > 0.05 for all timepoints) and thermal (**H**, radiant heat paw withdrawal, Vehicle + CCI-779 versus A-443654 + CCI-779 Day 1 q(8.733) = 5.540, $p = 0.009$, Day 3 q(15.89) = 3.872, $p = 0.036$; Vehicle + CCI-779 versus A-443654 + Vehicle Day 1 q(13.59) = 5.078, $p = 0.008$, Day 2 q(13.26) = 3.848, $p = 0.042$; A-443654 + CCI-779 versus A-443654 + Vehicle, p > 0.05 for all timepoints) hypersensitivity. Two-way ANOVA followed by Tukey's post-hoc comparison (**G-H**). All data are presented as mean ± s.e.m. *$p < 0.05$, **$p < 0.01$, ***$p < 0.001$, ****$p < 0.0001$. ns, not significant.

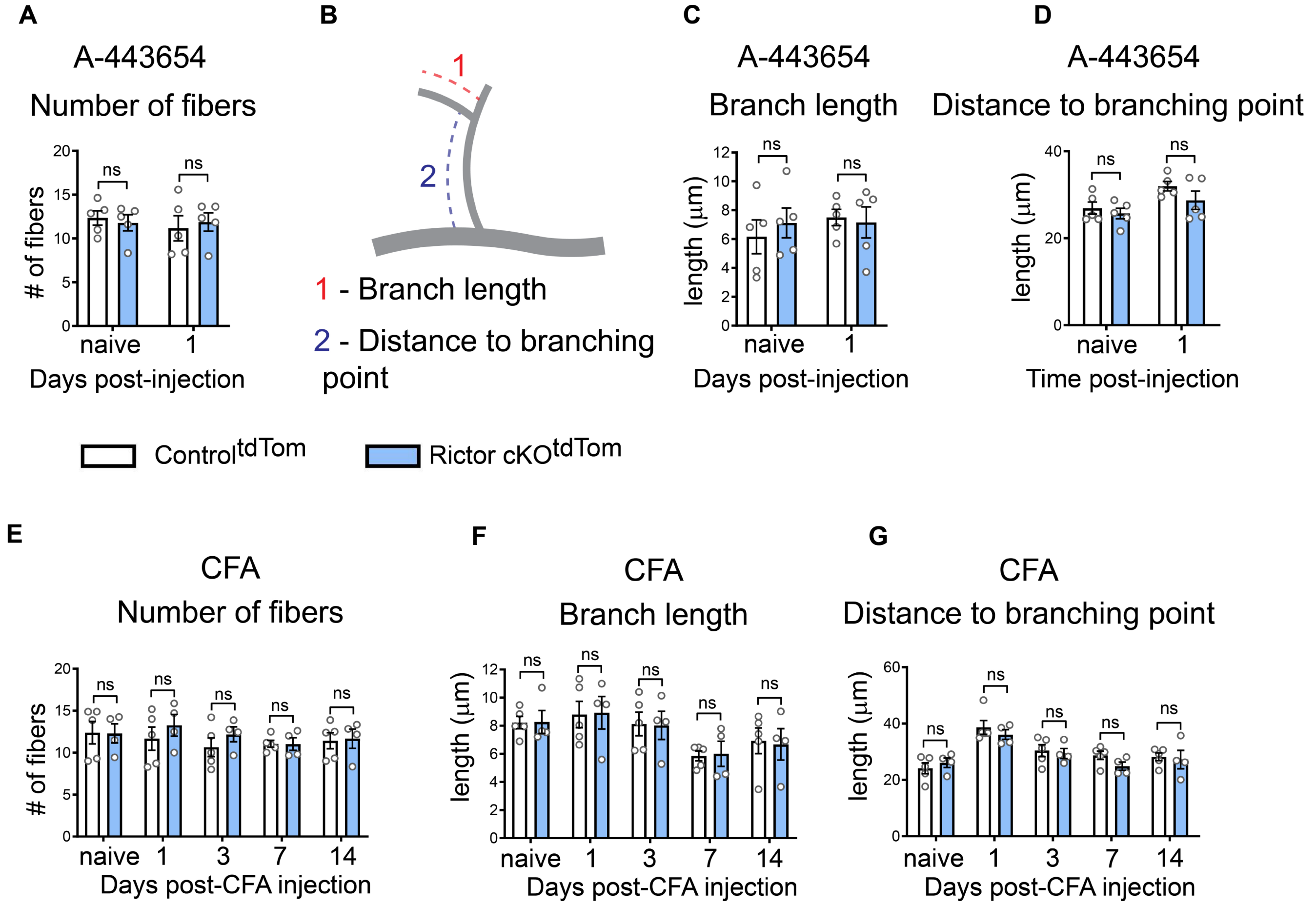


**Supplemental Figure 4. mTORC2 activation and inflammation does not affect the number of nociceptive fibers in epidermis.** A-443654 intraplantar injection (20 µL, 8 mM) does not change the number of nociceptive fibers (**A**), branch length (**C**) and distance to branching point (**D**) in glabrous skin of Control$^{tdTom}$ and Rictor cKO$^{tdTom}$ mice 24 hours post-injection. (**B**) Schematic shows how branch length and distance to branching point were calculated. CFA intraplantar injection does not affect the number of nociceptive fibers (**E**), branch length (**F**) and distance to branching point (**G**) in glabrous skin of Control$^{tdTom}$ and Rictor cKO$^{tdTom}$ mice 24 hours post-injection. Two-way ANOVA followed by Bonferroni's post-hoc comparison. All data are presented as mean ± s.e.m. ns, not significant.

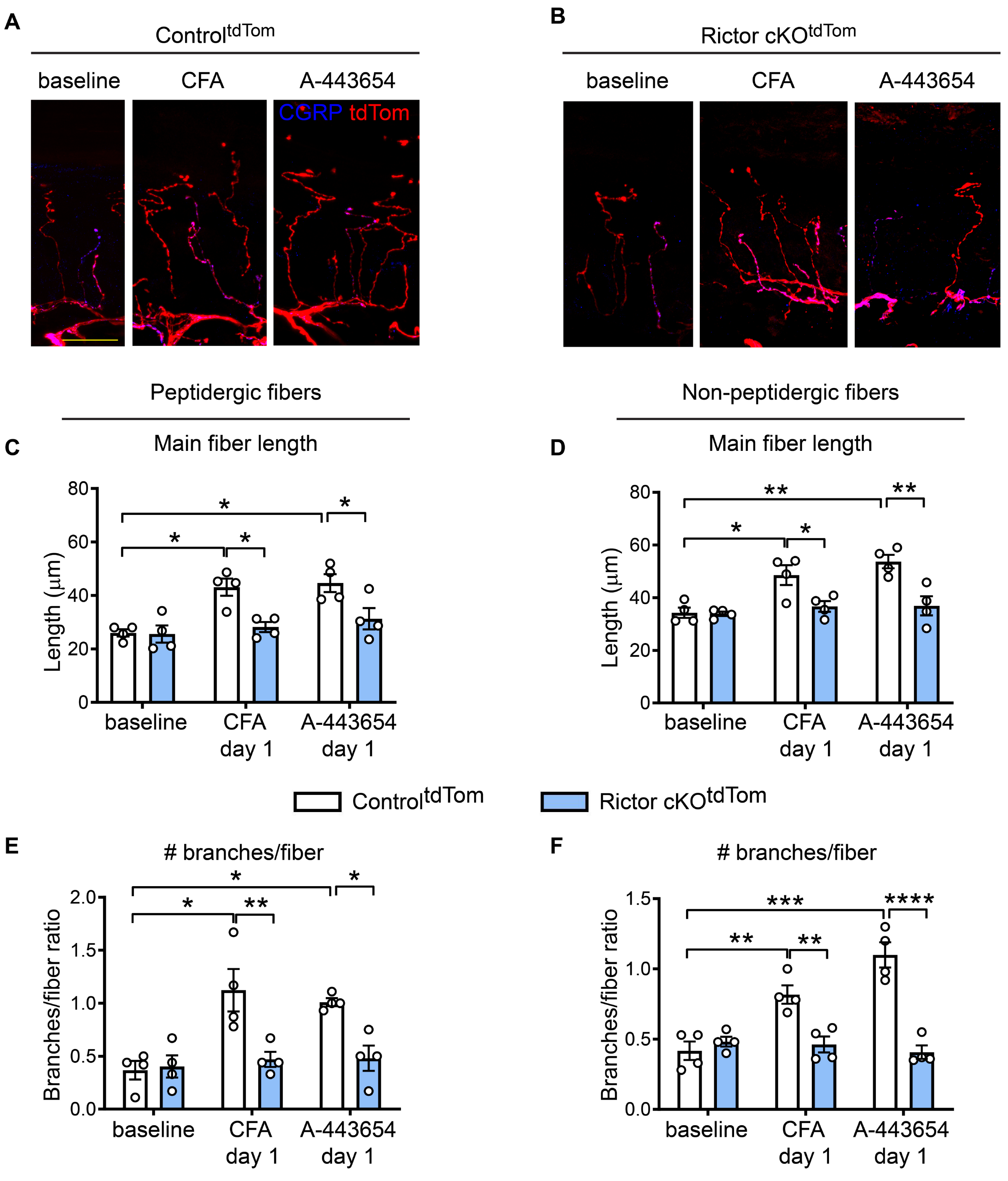


**Supplemental Figure 5. Morphological changes following CFA and mTORC2 activation are present in both peptidergic and non-peptidergic nociceptors.** Control$^{tdTom}$ (**A**) and Rictor cKO$^{tdTom}$

(**B**) mice were injected intraplantar with CFA and A-443654 and images of tdTomato in glabrous skin tissue, co-stained with anti-CGRP antibody, were collected 24 hours post-injection. Quantification revealed that CFA and A-443654 increase main fiber length of peptidergic (**C**) and non-peptidergic (**D**) nociceptors in epidermis in Control$^{tdTom}$ but not Rictor cKO$^{tdTom}$ mice (n = 4 mice per group). Similarly, CFA and A-443654 increased the number of branches per fiber of peptidergic (**E**) and non-peptidergic (**F**) nociceptors in Control$^{tdTom}$ but not Rictor cKO$^{tdTom}$ mice. Two-way ANOVA followed by Bonferroni's post-hoc comparison. All data are presented as mean ± s.e.m. *$p < 0.05$, **$p < 0.01$, ***$p < 0.001$, ****$p < 0.0001$. ns, not significant. Scale bar, 10 µm.

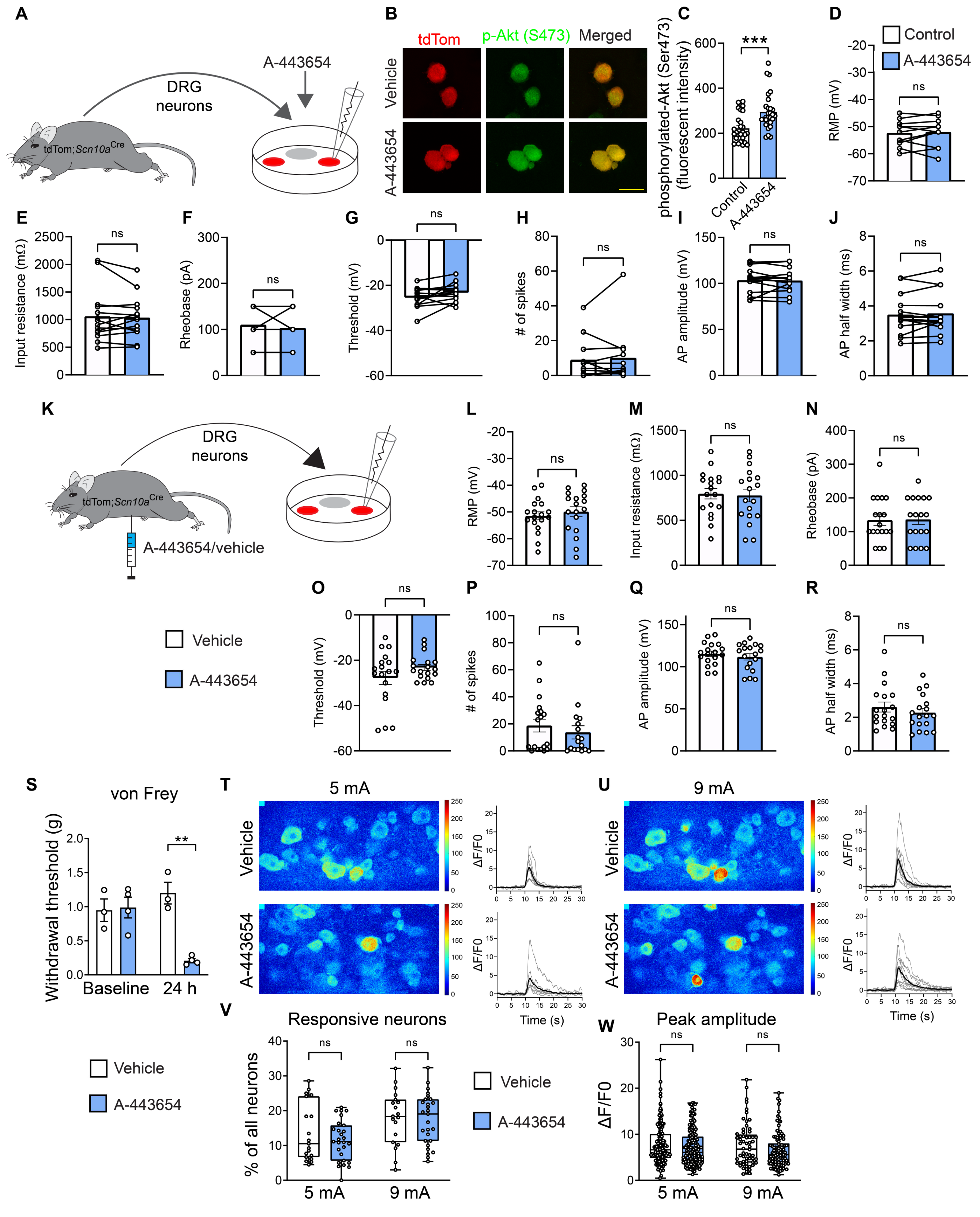


**Supplemental Figure 6. Activation of mTORC2 does not affect intrinsic excitability of nociceptors.**

(**A**) Lumbar DRGs from tdTomato;*Scn10a*$^{Cre}$ mice were cultured and 24 h later treated with saline or A-

443654 (1 μM for 10 minutes). (**B**) Immunostained for p-Akt (S473). Scale bar, 20 μm. (**C**) Quantification of p-Akt (S473) in nociceptor (tdTomato$^+$) (n = 25-28 cells from 3 animals/group, student's t-test). No differences were detected in resting membrane potential (**D**), input resistance (**E**), rheobase (**F**), AP threshold (**G**), number of spikes (**H**), AP amplitude (**I**), and AP half width (**J**) in nociceptors (tdTomato$^+$) from tdTomato;*Scn10a$^{Cre}$* mice before (control) and after application of A-443654 (n = 14 cells from 6 animals/group). **(K)** tdTomato;*Scn10a$^{Cre}$* mice were injected with A-443654 (2.5 mg/kg) or vehicle (saline), and 24 h later lumbar DRGs were cultured. Recordings from nociceptors (tdTomato$^+$) 24 h after culturing showed no differences in resting membrane potential (**L**), input resistance (**M**), rheobase (**N**), AP threshold (**O**), number of spikes (**P**), AP amplitude (**Q**), and AP half width (**R**) between vehicle- and A-443654-injected mice (n = 18 cells from 5 animals/group). (**S**) Prior to performing in vivo calcium imaging from DRG nociceptors, mechanical sensitivity of *Scn10a$^{Cre}$*;GCaMP6s mice was assessed in von Frey test at the baseline and 24 h post-A-443654 administration (2.5 mg/kg, i.p.) (n = 3-4, two-way ANOVA with Bonferroni's post-hoc analysis). Representative color-coded heatmap of imaging fields showing neuronal responses to moderate (**T**, 5 mA) and strong (**U**, 9 mA) electrical stimulation of the sciatic nerve in vehicle- and A-443654-injected mice, and corresponding $Ca^{2+}$ curves (right). The gray traces are the $Ca^{2+}$ transients from individual nociceptors, while the black trace is the average of all the responsive neurons from the same imaging field. The color scale indicates maximum $\Delta F$. (**V**) No difference was found in the percentage of neurons in each imaging field that responded to different intensity of electrical stimuli in vehicle- and A-443654-injected mice (number of imaged fields: n = 19 from 3 mice for vehicle and n = 27 from 4 mice for A-443654). (**W**) No difference was found in the peak amplitude in response to different intensity of electrical stimulation in vehicle- and A-443654-injected mice. For **V-W**, the box shows quartiles, and whiskers show minimum–maximum. All other data are presented as mean ± s.e.m. $^{**}p < 0.01$, ns, not significant.

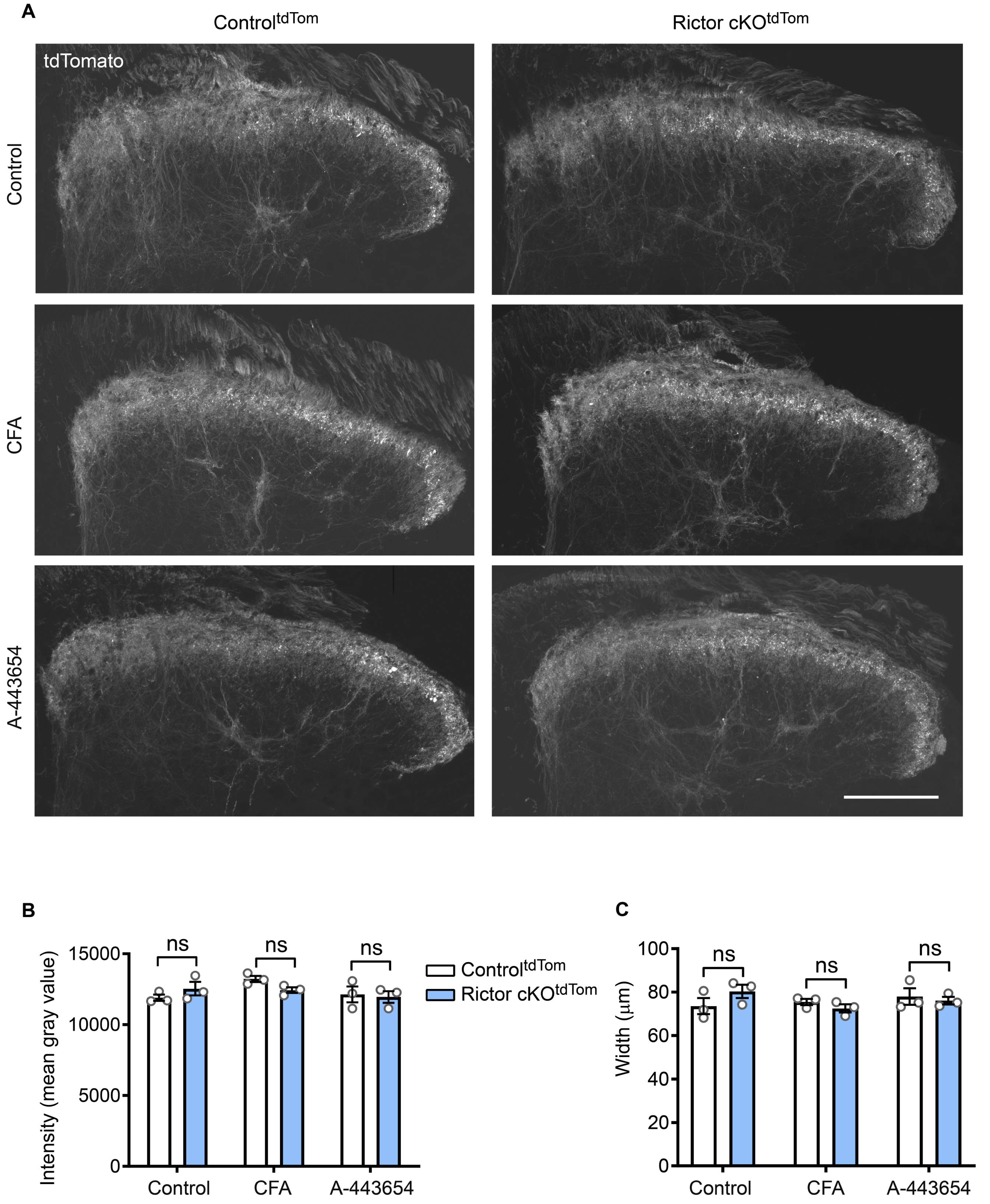


**Supplemental Figure 7. No macroscopic changes in central branch of nociceptors following inflammation and mTORC2 activation.** Control$^{tdTom}$ and Rictor cKO$^{tdTom}$ mice were injected with CFA

(intraplantar) and A-443654 (intraplantar) and spinal cord sections were prepared 24 hours post-injection and imaged using confocal microscopy with AiryScan. Representative images (**A**) and quantification show no macroscopic differences in the intensity (**B**) and tdTomato signal distribution width (**C**) in the spinal cord (two-way ANOVA followed by Bonferroni's post-hoc comparison). All data are presented as mean ± s.e.m. ns, not significant. Scale bar, 150 μm.

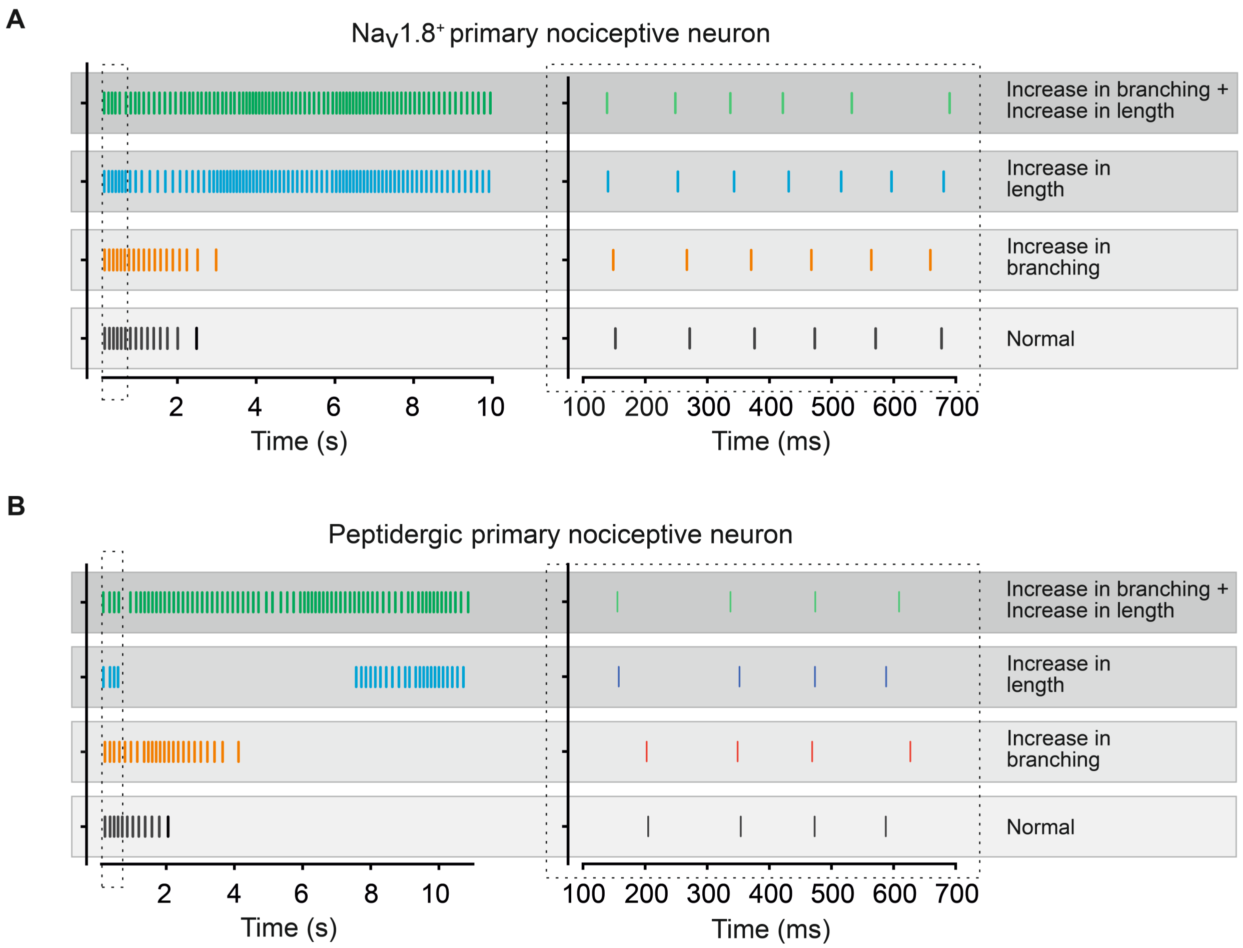


**Supplemental Figure 8**. **Structural changes in distal nociceptive endings alter AP firing pattern. (A)** A spike raster plot showing the AP timing following stimulation of all $Na_v1.8$-positive nociceptor terminal branches with a capsaicin-like current, in normal conditions (black), simulated increase in branching (orange); simulated increase in length of the terminal endings (blue) and a combined increase in branching and terminal length (green). **(B)** Same as (A) but following stimulation of all $Na_v1.8$-positive peptidergic nociceptor terminal branches with a capsaicin-like current, in normal conditions (black), simulated increase in branching (orange); simulated increase in length of the terminal endings (blue) and a combined increase in branching and terminal length (green). Inset shows an expanded x-axis.

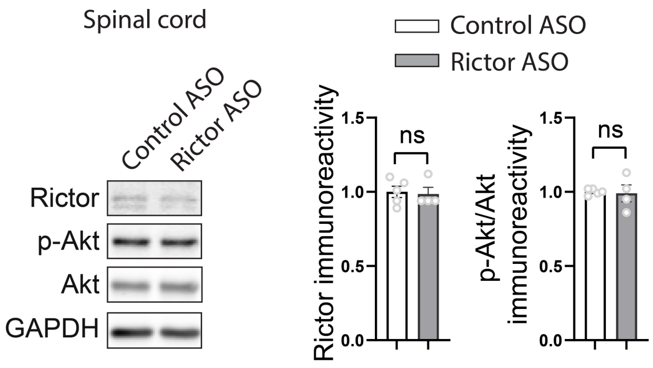


**Supplemental Figure 9. Injection of Rictor-ASO subcutaneously does not affect Rictor protein level in the spinal cord.** No differences in Rictor or p-Akt protein levels between animals injected with Control- or Rictor-ASO in spinal cord lysates. Student’s t-test. All data are presented as mean ± s.e.m. ns, not significant.
